# A peroxisomal heterodimeric enzyme is involved in benzaldehyde synthesis in plants

**DOI:** 10.1038/s41467-022-28978-2

**Published:** 2022-03-15

**Authors:** Xing-Qi Huang, Renqiuguo Li, Jianxin Fu, Natalia Dudareva

**Affiliations:** 1 Department of Biochemistry, Purdue University, West Lafayette, IN USA; 2 Purdue Center for Plant Biology, Purdue University, West Lafayette, IN 47907 USA; 3 Department of Horticulture and Landscape Architecture, Purdue University, West Lafayette, IN USA; 4 Present Address: School of Landscape Architecture, Zhejiang Agriculture & Forestry University, 311300 Hangzhou, P.R. China

**Keywords:** Secondary metabolism, Plant molecular biology, Biosynthesis

## Abstract

Benzaldehyde, the simplest aromatic aldehyde, is one of the most wide-spread volatiles that serves as a pollinator attractant, flavor, and antifungal compound. However, the enzyme responsible for its formation in plants remains unknown. Using a combination of in vivo stable isotope labeling, classical biochemical, proteomics and genetic approaches, we show that in petunia benzaldehyde is synthesized via the β-oxidative pathway in peroxisomes by a heterodimeric enzyme consisting of α and β subunits, which belong to the NAD(P)-binding Rossmann-fold superfamily. Both subunits are alone catalytically inactive but, when mixed in equal amounts, form an active enzyme, which exhibits strict substrate specificity towards benzoyl-CoA and uses NADPH as a cofactor. Alpha subunits can form functional heterodimers with phylogenetically distant β subunits, but not all β subunits partner with α subunits, at least in Arabidopsis. Analysis of spatial, developmental and rhythmic expression of genes encoding α and β subunits revealed that expression of the gene for the α subunit likely plays a key role in regulating benzaldehyde biosynthesis.

## Abstract (web summary)

Benzaldehyde is a simple aromatic aldehyde that attracts pollinators, has antifungal properties and contributes to flavor in many plants. Here the authors show that benzaldehyde is synthesized in petunia via the benzoic acid β-oxidative pathway by a peroxisomal heterodimeric enzyme consisting of α and β subunits.

## Introduction

Benzaldehyde is the simplest aromatic aldehyde found in nature, consisting of a single benzene ring bearing an aldehyde group. Phylogenetically, it is one of the most widely distributed volatiles and is likely the most ancient compound given that it is produced not only by over 50% of plant families analyzed so far for their volatile profiles^1^, but also by insects and non-insect arthropods^2^. Benzaldehyde plays important roles in chemical communications serving as a sex, aggregation and alarm pheromone as well as a defense compound in some insects and non-insect arthropods, and as a pollinator attractant, flavor volatile and antifungal compound in plants^2^. Found in scents of numerous flowers, benzaldehyde is readily detected by hawk moths eliciting strong responses from their antennas^3,4^. Moreover, the loss of its emission led to a shift in reproductive strategy in the genus *Capsella* from pollination by insects to self-fertilization^5^.

Benzaldehyde possesses a characteristic pleasant almond-like odor and contributes to aromas of many fruits including cherry, peach, cranberry, raspberry, and melon^6,7^. However, when emitted by postharvest tomato fruits it also inhibits *Botrytis cinerea* infection thus preventing gray mold disease, which causes economic losses in tomato fruit industries worldwide^8^. In addition, benzaldehyde is present in almonds, apricots, apples, and cherry kernels as a diglucoside, amygdalin, from which it can be released by hydrolysis along with a toxic byproduct hydrogen cyanide^9^. Long known for its smell and taste, benzaldehyde is the most important, after vanillin, contributor to the flavor industry^7^. It is of economic value to the cosmetic and fragrance industries and is used extensively as a precursor to plastic additives and some dyes. To date, benzaldehyde is primarily produced synthetically by air oxidation of toluene^10^. Natural benzaldehyde, which constitutes ~1.5% of total annual world production, is mainly obtained by retroaldol reaction of natural cinnamaldehyde extracted from cassia oil^11^. Although the natural benzaldehyde market is growing every year, we still do not know how this simple aromatic compound is synthesized in plants.

**Fig. 1:**
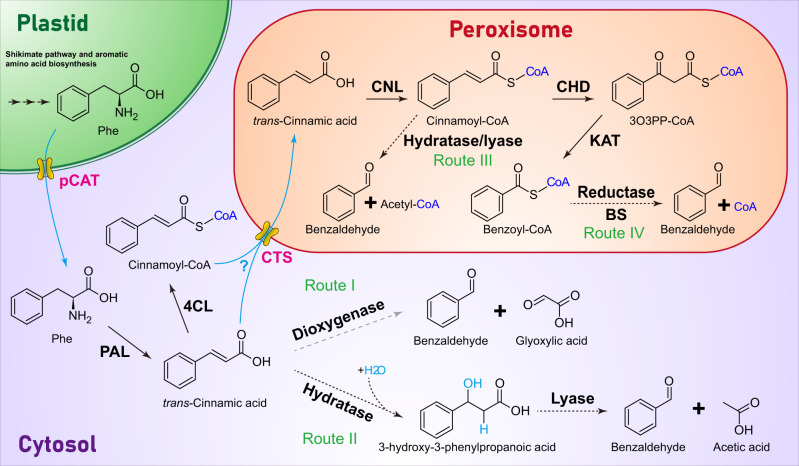
Proposed pathways for benzaldehyde biosynthesis in plants. Enzymes responsible for each biochemical reaction are shown in bold black. Solid black arrows show biochemical steps with their encoding genes discovered, dashed black arrows show steps established only at enzyme level and dashed gray arrow presents putative step. Cinnamoyl-CoA formation is also shown in the cytoplasm since 4CL enzymes capable of activating *trans*-cinnamic acid were reported in some plants^19,31^. Transporters involved in metabolite transport between different subcellular locations are in bold magenta, with the flow of metabolites indicated by cyan arrows. CHD cinnamoyl-CoA hydratase/dehydrogenase, CNL cinnamate-CoA ligase, CTS peroxisomal cinnamic acid/cinnamoyl-CoA transporter COMATOSE. The substrate specificity of CTS is unclear, as indicated by a cyan question mark; KAT 3-ketoacyl thiolase, PAL phenylalanine ammonia lyase, pCAT plastidial cationic amino acid transporter, Phe phenylalanine, 3O3PP-CoA 3-oxo-3-phenylpropanoyl-CoA.

Like most phenylpropanoid/benzenoid compounds, benzaldehyde biosynthesis begins with the deamination of phenylalanine to *trans*-cinnamic acid by the well-known enzyme phenylalanine ammonia lyase^12,13^. The following conversion of cinnamic acid to benzaldehyde requires (i) shortening of the side chain by two carbons and (ii) introduction of the aldehyde functional group to the side chain, which may occur as an integral part of chain shortening. Several routes (summarized in Fig. 1) have been proposed for benzaldehyde formation from cinnamic acid including β-oxidative and non-β-oxidative pathways, the latter of which could be CoA-dependent or CoA-independent. In the CoA-independent non-β-oxidative pathway, benzaldehyde originates from cinnamic acid by the direct cleavage of the double bond by a putative dioxygenase analogous to partially purified and characterized *Vanilla planifolia* phenylpropanoid 2,3-dioxygenase, which produces an aldehyde vanillin and glyoxylic acid from ferulic acid^14^ (Fig. 1, Route I). Alternatively, the double bond in the side chain of cinnamic acid may undergo hydration to form 3-hydroxy-3-phenylpropanoic acid intermediate before the cleavage by a hydratase/lyase-type enzyme yielding benzaldehyde and acetate (Fig. 1, Route II). Enzyme activity catalyzing similar non-oxidative formation of benzaldehyde from cinnamic acid has been recently reported in cell culture of Asian pear *Pyrus pyrifolia*^15^, however no gene encoding this enzyme has been isolated.

Despite multiple potential non-β-oxidative routes for benzaldehyde biosynthesis, feeding experiments with stable isotope-labeled (^2^H_6_,^18^O)3-hydroxy-3-phenylpropanoic acid supported the existence of a β-oxidative route in cucumber (*Cucumis sativus*) and *Nicotiana attenuata*^16^. Moreover, recent genetic studies have suggested that benzaldehyde biosynthesis in plants depends on a cinnamoyl-CoA intermediate produced by peroxisomal cinnamate-CoA ligase (CNL)^5,17,18^, which catalyzes the first committed step in benzoic acid biosynthesis via the β-oxidative pathway^19–21^ and CoA-dependent non-β-oxidative pathway^22,23^. Indeed, loss-of-function mutations in *CNL* led to the loss of benzaldehyde emission in bird-pollinated *Petunia exserta*^18^ and selfing *Capsella rubella*^5,24^. Cinnamoyl-CoA could be converted directly to benzaldehyde by an enoyl-CoA hydratase/lyase (Fig. 1, Route III) thus linking the β-oxidative and non-β-oxidative pathways. While a protein fraction with an enoyl-CoA hydratase/lyase activity catalyzing the hydration and non-oxidative cleavage of cinnamoyl-CoA to benzaldehyde (probably through an enzyme-bound 3-enoyl-CoA intermediate) has been characterized from *Hypericum androsaemum* cell culture^25^, the corresponding gene still remains unknown. In the proposed β-oxidative route, cinnamoyl-CoA in peroxisomes is first converted to benzoyl-CoA by cinnamoyl-CoA hydratase/dehydrogenase (CHD) and 3-ketoacyl-CoA thiolase (KAT)^21^ followed by reduction to benzaldehyde by an enzyme similar to cinnamoyl-CoA reductase (CCR), which catalyzes the reduction of hydroxycinnamoyl-CoA thioesters to their corresponding aldehydes in lignin biosynthesis (Fig. 1, Route IV)^26^. The CCR enzymes typically exhibit broad substrate specificity and utilize *p*-coumaroyl-CoA, caffeoyl-CoA, feruloyl-CoA, 5-hydroxyferuloyl-CoA and sinapoyl-CoA^27^. Benzoyl-CoA itself is a poor substrate for most of the characterized plant CCR isoforms with exception of three out of 18 CCR family members in cucumber *Cucumis sativus*^28^. However, no benzoyl-CoA specific reductase has been reported yet.

To elucidate benzaldehyde biosynthesis *in planta*, we used *Petunia hybrida* cv. Mitchell flowers, which produce high levels of benzaldehyde^12,29^, as a model system. By combining in vivo stable isotope labeling with classical biochemical, proteomics and genetic approaches, we found that benzaldehyde is synthesized via the β-oxidative pathway and a heterodimeric enzyme consisting of α and β subunits, both of which belong to NAD(P)-binding Rossmann-fold superfamily, is responsible for its formation. We also showed that this enzyme exhibits strict substrate specificity towards benzoyl-CoA and uses NAPDH as a cofactor. Analysis of tissue-specific, developmental, and rhythmic expression of genes encoding α and β subunits revealed that expression of the gene for the α subunit correlates with benzaldehyde emission and likely plays a key role in regulating of benzaldehyde biosynthesis. Moreover, peroxisomal localization of both subunits suggests that benzaldehyde biosynthesis via the β-oxidative route takes place in peroxisomes.

## Results

### Benzaldehyde is synthesized via the β-oxidation pathway in petunia

**Fig. 2:**
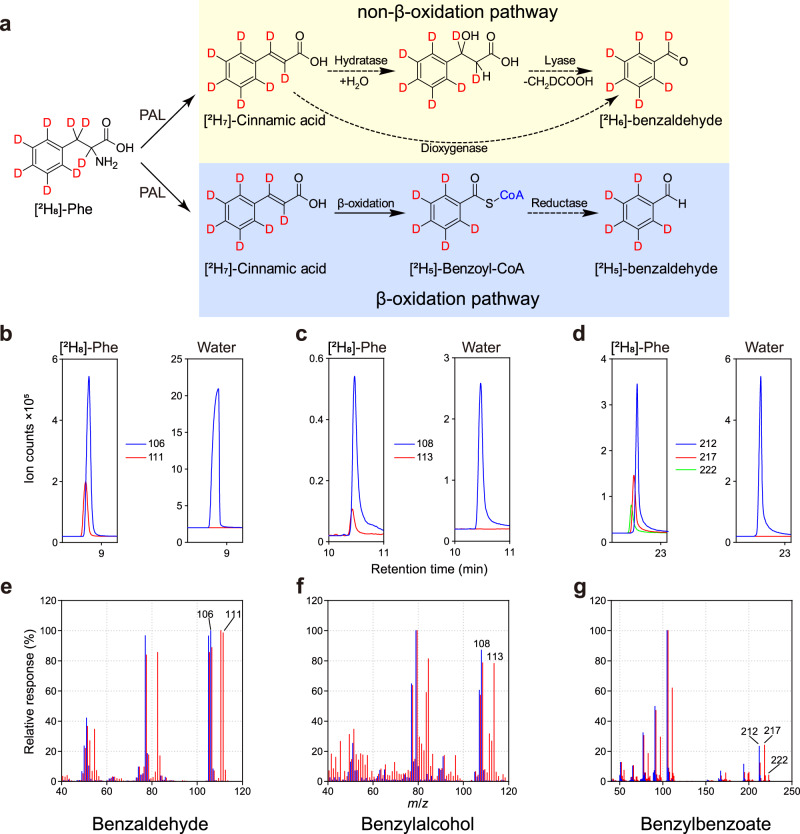
Predicted and in vivo labeling of benzaldehyde synthesized from [^2^H_8_]-Phe in petunia petals. **a** Two hypothetical routes for biosynthesis of benzaldehyde and its predicted labeling from [^2^H_8_]-Phe. Established biochemical reactions are presented by solid arrows, while unidentified steps are shown by dashed arrows. PAL, phenylalanine ammonia lyase. **b**–**d** GC–MS chromatogram of benzaldehyde (**b**), benzylalcohol (**c**) and benzylbenzoate (**d**) produced by petunia petals fed with [^2^H_8_]-Phe for 2 h presented as extracted ion current of indicated *m*/*z*. Unlabeled compounds are shown by blue lines. **e**–**g** Overlays of unlabeled (blue lines) and newly synthesized from [^2^H_8_]-Phe labeled (red lines) mass spectra for benzaldehyde (**e**), benzylalcohol (**f**) and benzylbenzoate (**g**) shown in (**b**–**d**). The response of the most abundant peak in each graph was set to 100%.

To determine whether benzaldehyde biosynthesis occurs via the β-oxidative or non-β-oxidative pathway, petunia corollas were fed with L-[^2^H_8_]-Phe for 2 h followed by collection of floral volatiles from 18:00 till 22:00, the time of the day with the highest scent emission^12^. Formation of benzaldehyde via the non-β-oxidative route is expected to keep the label at the aldehyde position of the product resulting in benzaldehyde molecules +6 atomic mass units larger (Fig. 2a). However, benzaldehyde was labeled by +5 atomic mass units suggesting that its biosynthesis instead proceeds via the β-oxidative pathway, which is shortening the propyl side chain of phenylalanine products by two carbon units leaving deuterium atoms only on the benzene ring (Fig. 2b, e and Table 1). Similarly, benzylalcohol was labeled by +5 atomic mass units (Fig. 2c, f and Table 1), indicating that it also originates from the β-oxidative pathway and further supporting our previous results about the existence of interconversion between benzylalcohol and benzaldehyde^12^. The lower labeling of benzylalcohol relative to benzaldehyde is due to its dilution by the large internal pool of unlabeled benzylalcohol that exists in petunia flowers^30^. Since benzoyl-CoA is not only an immediate precursor of benzaldehyde in the β-oxidative pathway, but also one of the precursors of benzylbenzoate, the labeling pattern of the latter was analyzed. Indeed, both benzoyl-CoA and benzylalcohol moieties were labeled by +5 atomic mass units in benzylbenzoate (Fig. 2d, g and Table 1).

**Table 1:** Labeling of VOCs after feeding of deuterium labeled phenylalanine and benzoic acid.

Labeled compounds	^2^H_8_-Phenylalanine (%)	^2^H_5_-Benzoic acid (%)
Benzaldehyde (D5, 111)	22.51 ± 5.45	23.03 ± 2.20
Benzylalcohol (D5, 113)	12.87 ± 3.36	12.37 ± 1.03
Phenylacetaldehyde (D7 + D8, 97 + 98)	29.76 ± 8.26	n.d.
Methylbenzoate (D5, 110)	25.69 ± 4.65	71.45 ± 3.44
Phenylethanol (D7, 98)	22.32 ± 7.34	n.d.
Eugenol (D5, 169)	5.79 ± 1.84	n.d.
Vanillin (D4, 156)	4.41 ± 1.05	n.d.
Isoeugenol (D5, 169)	4.94 ± 0.94	n.d.
Benzylbenzoate (D5, 217)	11.92 ± 1.90	24.33 ± 4.09
Benzylbenzoate (D10, 222)	2.88 ± 0.64	9.97 ± 3.53
Benzoyl moiety of Benzylbenzoate (105)	7.76 ± 1.12	22.88 ± 5.54
Alcohol moiety of Benzylbenzoate (91)	10.80 ± 2.31	21.86 ± 4.98

Shown are percentage of labeling, means ± SE, *n* = 3 biological replicates; n.d., not detected. The data were calculated based on peak areas integrated from extracted ion current (EIC) chromatograms of labeled (number of additional mass units, *m*/*z* used for calculation, as indicated in the brackets) and unlabeled compounds.

To further examine precursor-product relationships, ^2^H_5_-benzoic acid was supplied to excised petunia corollas for 2 h and floral volatiles were analyzed for their isotopic abundances. Benzoic acid can be activated to benzoyl-CoA by cinnamate:CoA ligases-like enzymes^19,31^, thus providing potential precursors for benzaldehyde and subsequently benzylalcohol syntheses. Both benzaldehyde and benzylalcohol were labeled in these experiments along with benzylbenzoate (Table 1), which relies on benzoyl-CoA derived predominantly from the β-oxidative pathway^32^.

**Fig. 3:**
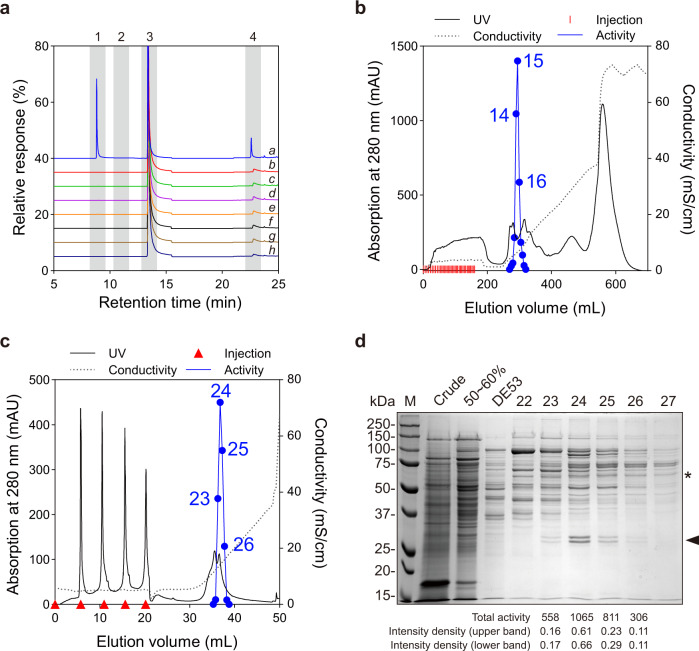
Partial purification of native PhBS from petunia flowers. **a** GC–MS analysis of BS activity in petunia flower crude protein extracts. Substrates and conditions for each reaction are as follows: (a) crude extracts + benzoyl-CoA + NADPH; (b) crude extracts + benzoyl-CoA + NADP^+^; (c) crude extracts + benzoyl-CoA + NADH; (d) crude extracts + benzoyl-CoA + NAD^+^; (e) crude extracts + benzoyl-CoA; (f) crude extracts + benzoic acid + NADPH; (g) heat denatured crude extracts + benzoyl-CoA + NADPH; (h) crude extracts + cinnamic acid. Compounds labeled with numbers are: (1) benzaldehyde, (2) benzylalcohol, (3) internal standard (naphthalene), and (4) benzylbenzoate. The response of internal standard in each run was set as 100%. **b** DE53 anion exchange chromatography of BS activity in petunia crude extracts. Fractions with BS activity are numbered. **c** MonoQ anion exchange chromatography of combined BS activity containing fractions 14 and 15 from DE53 chromatography. Fractions with profound BS activity are numbered. **d** SDS–PAGE analysis of purification steps for PhBS. Active fractions from successive purification steps were run on 12% SDS–PAGE. The indicated lanes correspond: Crude, petunia flower crude protein extract (~40 µg); 50–60%, proteins precipitated at 50–60% ammonium sulfate saturation (~40 µg); DE53, combined fractions 14 and 15 after DE53 chromatography (~20 µg); and 22–27, fractions separated by MonoQ chromatography shown in (**c**) (10 μL each). Asterisk (*) indicates the size of native PhBS (around 60 kDa); triangle indicates the position of two closely migrated bands representing PhBSα and PhBSβ. Total BS activities (pKat) and intensity density data of both bands are listed below the gel for fractions 23–26. The partial purification and SDS–PAGE analysis were repeated three times with similar results.

Since in vivo labeling experiments with petunia petals suggested that benzoyl-CoA serves as a benzaldehyde precursor, we next tested for the presence of benzoyl-CoA reductase activity *in planta*. Crude protein extracts from petunia petals collected around the peak of emission were incubated with several different assay mixtures containing benzoyl-CoA and different reducing cofactors. In these experiments, efficient conversion of benzoyl-CoA to benzaldehyde was observed only in the presence of NADPH (Fig. 3a). No benzaldehyde was formed when benzoyl-CoA was replaced by benzoic acid or cinnamic acid or when NADH, NADP^+^, NAD^+^ were supplied instead of NADPH in reaction with benzoyl-CoA. Controls lacking NADPH or containing denatured crude extract produced no detectable product (Fig. 3a). Since petunia petals contain a large internal pool of benzylbenzoate, this compound was detected in all reactions despite using desalted proteins in assays. However, its level increased only upon incubation of crude protein extracts with benzoyl-CoA and NADPH, suggesting that part of the produced benzaldehyde was reduced to benzylalcohol, which was then rapidly converted to benzylbenzoate in the presence of excessive benzoyl-CoA amount through the action of benzoyl-CoA:benzylalcohol/2-phenylethanol benzoyltransferase (BPBT)^12,33^. Taken together, these results suggest that benzaldehyde is synthesized via the β-oxidative pathway and a putative benzaldehyde synthase catalyzes the NADPH-dependent reduction of benzoyl-CoA to the corresponding aldehyde.

### Partial purification and identification of benzaldehyde synthase

Petunia genome contains two *CCR* genes, out of which *PhCCR1* is highly expressed in scent-emitting flower tissues^34^. Biochemical characterization of the corresponding enzyme revealed that it is most active with feruloyl-CoA, followed by sinapoyl-CoA and *p*-coumaroyl CoA, and possesses a very low activity with caffeoyl-CoA and benzoyl-CoA^27^. Moreover, the lack of changes in the benzoyl-CoA pool size and benzaldehyde emission upon 90% RNAi suppression of *PhCCR1* expression^34^ suggests that efficient benzaldehyde formation by petunia petals and detected benzaldehyde synthase activity (Fig. 3a) respectively relies on and belongs to the enzyme other than PhCCR1. Thus, benzaldehyde synthase was partially purified using ammonium sulfate precipitation, DE53 anion exchange (Fig. 3b) and Mono Q chromatography (Fig. 3c). This purification protocol resulted in up to 80.5-fold increase in specific activity over the crude extract, with a recovery of 3.8% (Table 2). Starting with 60 g of fresh 2-day-old corolla tissue, four fractions with specific activities ranging from 2748 to 5336 pKat mg protein^−1^ were obtained after Mono Q anion-exchange chromatography (Table 2). The apparent molecular mass of native plant benzaldehyde synthase was determined by size-exclusion chromatography on a Superose 12 10/300 column and appeared to be 58 kDa (Supplementary Fig. 1). However, when four Mono Q fractions with the highest specific activities were analyzed on SDS–PAGE, no proteins with size between 50 and 75 kDa displayed a positive correlation with benzaldehyde synthase activities in the various fractions. Instead, intensities of two closely migrating bands around 30 kDa well correlated with benzaldehyde synthase activities (Fig. 3d). The sum of molecular masses of these two bands calculated from their migration in SDS–PAGE gel was close to that of native plant benzaldehyde synthase determined by gel filtration chromatography. Moreover, gel filtration chromatography of the Mono Q fraction 24, which contained the highest amounts of the two proteins, also revealed a strong positive correlation between the presence of the two bands and benzaldehyde synthase activities (Supplementary Fig. 2), suggesting that petunia benzaldehyde synthase might be a homodimer of either of the individual proteins or composed of two distinct subunits.

**Table 2:** PhBS purification from petunia petals.

Fraction	Total activity (pKat)	Protein content (mg)	Specific activity (pKat/mg)	Purification fold	Recovery (%)
Crude extract (60 g tissue)	72,533	1094.4	66.3	1.0	100.0
Ammonium sulfate (50–60%)	23,680	199.7	118.6	1.8	32.7
DE53-14	3167	2.33	1359	20.5	4.4
DE53-15	4246	2.38	1788	27.0	5.8
MonoQ-23	558	0.203	2748	41.5	0.8
MonoQ-24	1065	0.232	4589	69.2	1.5
MonoQ-25	811	0.152	5336	80.5	1.1
MonoQ-26	306	0.086	3559	53.7	0.4

Fractions 24–26 from Mono Q chromatography as well as fraction 24 from gel filtration chromatography of the Mono Q fraction 24 (SEC 24) were subjected to proteomic analysis. Peptides obtained by UPLC-QTOF MS/MS were searched against the protein sequences encoded by *Petunia axillaris* genome, with NAD(P)-binding proteins being of a particular interest. A total of 744 proteins were identified from all analyzed fractions (Supplementary Data 1), with only Peaxi162Scf00811g00011 and Peaxi162Scf00776g00122 encoding proteins around 30 kDa in fraction SEC 24. Since fraction SEC 24 contained no other NAD(P)-binding proteins despite the highest benzaldehyde synthase activity among all gel filtration fractions, we hypothesized that one or both of these two proteins are responsible for the benzaldehyde synthase activity. Therefore, based on results presented below, the slightly larger protein encoded by Peaxi162Scf00811g00011 we designated as a *Petunia hybrida* alpha subunit of benzaldehyde synthase (PhBSα), and the smaller protein encoded by Peaxi162Scf00776g00122 as a beta subunit of benzaldehyde synthase (PhBSβ).

*PhBSα* and *PhBSβ* encode proteins of 30.5 and 29.6 kDa, respectively, which belong to NAD(P)-binding Rossmann-fold superfamily and exhibit 30.3% identity and 50.4% similarity to each other. Phylogenetic analysis of three petunia species, *Petunia hybrida* and its two parental lines *Petunia axillaris* and *Petunia inflata*, revealed that only a single gene exists in *Petunia* genus for the β-subunit, while the α-subunit gene has more homologs (Supplementary Fig. 3). When the phylogenetic tree was expanded to the whole Solanaceae family, it appeared that the α-subunit resides in a large clade containing 3-oxoacyl-(acyl-carrier-protein) reductase FabG-like proteins participating in fatty acid biosynthesis, while the β-subunit is a member of a more limited clade with unknown functions (Supplementary Fig. 4).

### Benzaldehyde synthase uses benzoyl-CoA to produce benzaldehyde in vitro

**Fig. 4:**
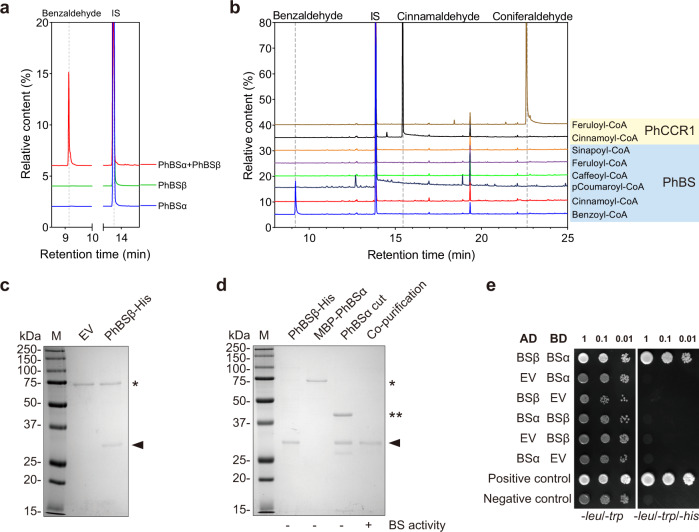
Heterodimeric nature and substrate specificity of PhBS. **a** GC–MS analysis of products formed by PhBSα and PhBSβ subunits, and their mixture at 1:1 ratio. The response of internal standard in each run was set as 100%. **b** GC–MS analysis of products formed by PhBS from different hydroxycinnamoyl-CoA substrates. GC-MS chromatograms of in vitro enzymatic assays using various substrates. Purified PhBS (1:1 ratio between α and β subunits) was incubated with benzoyl-CoA and its structural analogs including cinnamoyl-CoA, *para*-coumaroyl-CoA, caffeoyl-CoA, feruloyl-CoA, and sinapoyl-CoA. Formation of cinnamaldehyde and coniferaldehyde by purified PhCCR1 was used as a positive control. Shown are combined EICs of mass units 106 (benzaldehyde), 128 (internal standard), 131 (cinnamaldehyde), and 178 (coniferaldehyde). The response of internal standard in each run was set as 100%. IS, internal standard. **c** Pull-down analysis of PhBSβ-His binding to MBP-PhBSα. Purified MBP-PhBSα was incubated with bacterial lysate of pET32b empty vector (EV) or pET32b expressing PhBSβ-His, protein complex was purified using Amylose resin and analyzed by SDS–PAGE. (*) indicates the position of MBP-PhBSα; triangle indicates the position of PhBSβ-His. The experiment was repeated three times with similar results. **d** Pull-down analysis of PhBSα binding to PhBSβ-His. Purified MBP-PhBSα was digested with Factor Xa protease, incubated with purified PhBSβ-His, the complex was purified again using Ni-NTA column and analyzed by SDS-PAGE. (*) indicates the position of MBP-PhBSα; (**) indicates the position of free MBP tag; triangle represents the position of PhBSβ-His and untagged PhBSα, as indicated by BS activity. The experiment was repeated three times with similar results. **e** Y2H detection of PhBSα and PhBSβ interactions. Yeast cells harboring different combination of AD and BD were spotted at increasing dilutions on nonselective (-*leu*/-*trp*) and selective medium (-*leu*/-*trp*/-*his*). AD, activation domain of pGAD-T7 and BD, DNA-binding domain of pGBK-T7, to which petunia BSα and BSβ were fused; EV empty vector. At least 10 colonies from each combination were tested on selective medium with same results.

To determine the possible function of the proteins encoded by *PhBSα* and *PhBSβ*, the coding regions of both subunits were amplified from petunia petal cDNAs, subcloned into expression vectors, and expressed in *E. coli*. Neither of the two purified recombinant proteins (Supplementary Fig. 5a) displayed benzaldehyde synthase activity when tested alone in assay mixture containing benzoyl-CoA and NADPH (Fig. 4a). However, benzaldehyde was efficiently formed when the two subunit proteins were mixed in equal amounts (Fig. 4a). Further PhBS biochemical characterization revealed that the apparent *K*_m_ for benzoyl-CoA was 677.2 ± 80.5 μM and the catalytic efficiency (*k*_cat_/*K*_m_) was 13.9 ± 1.8 mM^−1^ s^−1^ (Table 3). These values lie within the range of catalytic efficiencies previously reported for other proteins (PAAS, PhIGS1, PhTE1) responsible for the formation of volatile benzenoid/phenylpropanoid compounds in petunia flowers^32,35,36^. The apparent *K*_m_ value for NADPH was 1342.0 ± 43.1 μM (Table 3).

**Table 3:** Kinetic parameters of recombinant BS from four species.

Organism	*K*_m_ (μM)	*V*_max_ (nKat/mg)	*k*_cat_ (s^−1^)^a^	*k*_cat_/*K*_m_ (mM^−1^ s^−1^)
*Petunia hybrida*^b^	677.2 ± 80.5	63.5 ± 2.7	9.39 ± 0.40	13.9 ± 1.8
*Petunia hybrida*^c^	1342.0 ± 43.1	67.1 ± 0.9	9.92 ± 0.14	7.4 ± 0.3
*Arabidopsis thaliana*^b^	242.8 ± 28.2	14.4 ± 0.8	2.12 ± 0.08	8.7 ± 1.1
*Prunus dulcis*^b^	629.4 ± 67.7	35.4 ± 1.3	5.26 ± 0.20	8.4 ± 1.0
*Solanum lycopersicum*^b^	767.3 ± 216.5	13.2 ± 3.0	1.96 ± 0.23	2.5 ± 0.8

Data are means ± error. Error values are standard errors as derived from nonlinear fit analyses. *n* = 3 technical replicates.

^a^Assuming one catalytic center per heterodimer of BS.

^b^*K*_m_ data were for benzoyl-CoA, measured at 4 mM NADPH.

^c^*K*_m_ data were for NADPH, measured at 3.6 mM benzoyl-CoA.

To analyze substrate specificity of PhBS, CoA esters were enzymatically synthesized from structural analogs of benzoic acid including cinnamic acid, *para*-coumaric acid, caffeic acid, ferulic acid and sinapic acid using purified recombinant petunia 4-coumarate:CoA ligase (Ph4CL1) (Supplementary Fig. 5b). A purified recombinant petunia PhCCR1 used as a positive control successfully reduced synthesized hydroxycinnamoyl-CoA thioesters to their respective aldehydes (Fig. 4b). However, no corresponding aldehyde production was detected when these CoA esters were incubated with PhBSα and PhBSβ (1:1 ratio) and NADPH (Fig. 4b). These results suggest that both subunits are required for activity of PhBS, which displays strict substrate selectivity for benzoyl-CoA.

### Benzaldehyde synthase is a peroxisomal heterodimeric enzyme

To examine whether the two subunits interact with each other forming a heterodimeric benzaldehyde synthase, we employed pull-down, yeast two-hybrid (Y2H) and bimolecular fluorescence complementation (BiFC) assays. For pull-down assays, PhBS was purified from a mixture of bacterial lysates containing maltose binding protein (MBP)-tagged α-subunit (MBP-PhBSα) and C-terminal His-tagged β-subunit (PhBSβ-His) using amylose resin. PhBSβ-His was co-purified with MBP-PhBSα as was evidenced by two bands visible on the SDS–PAGE (Fig. 4c). In addition, when untagged PhBSα was added to bacterial lysate containing His-tagged β-subunit, it was co-purified with PhBSβ-His on Ni-nitrilotriacetic acid (NTA) agarose. Since tag-free PhBSα and PhBSβ-His have the same apparent sizes, purified PhBS showed only one band on the SDS-PAGE but possessed benzaldehyde synthase activity indicating the presence of both subunits (Fig. 4d). Moreover, Y2H assays showed that the two subunits can directly interact with each other when PhBSα was fused to GAL4 DNA-binding domain (BD) and PhBSβ was fused to activation domain (AD). However, the negative growth response was observed when BD and AD were switched between two subunits probably due to a spatial blockage of the protein-protein interaction interface (Fig. 4e).

To analyze whether the two subunits can form heterodimers in vivo, BiFC was performed, which also allows detection of subcellular localization of interacting proteins^37^. Protein targeting prediction program WoLF PSORT predicted a possible peroxisomal localization for both subunits, despite neither contains the most common peroxisomal targeting sequence (PTS1)^38^. The coexpression of PhBSα tagged with the N-terminal part of enhanced yellow fluorescent protein (nEYFP) (nEYFP-PhBSα) with PhBSβ tagged with the C-terminal part of EYFP (cEYFP-PhBSβ) in *Nicotiana benthamiana* resulted in reconstituted fluorescent signal, which was detected in peroxisomes, confirming that PhBS is a peroxisomal heterodimeric enzyme (Supplementary Fig. 6a). To further verify the subcellular localization of PhBS subunits *in planta*, the coding region of each subunit was fused to either C-terminus of green fluorescent protein (GFP) or N-terminus of EYFP reporter gene and transiently co-expressed in *N. benthamiana* leaves with peroxisomal mCherry-labeled marker protein. The N-terminal fusion proteins, GFP-PhBSα and GFP-PhBSβ, showed peroxisomal localization while the C-terminal fusion proteins, PhBSα-EYFP and PhBSβ-EYFP, with blocked peroxisomal targeting signals failed to enter peroxisomes and accumulated in the cytosol instead (Supplementary Fig. 6b). Given the peroxisomal localization of PhBS, we also analyzed whether it could be involved in metabolism of short-chain fatty acyl-CoA esters. When purified PhBS was incubated with 1 mM of *n*-butanoyl-CoA, hexanoyl-CoA and crotonoyl-CoA, only trace amount of hexanal was detected with hexanoyl-CoA substrate (Supplementary Fig. 7).

### The expression of *PhBSα* and *PhBSβ* is differentially regulated in flowers

**Fig. 5:**
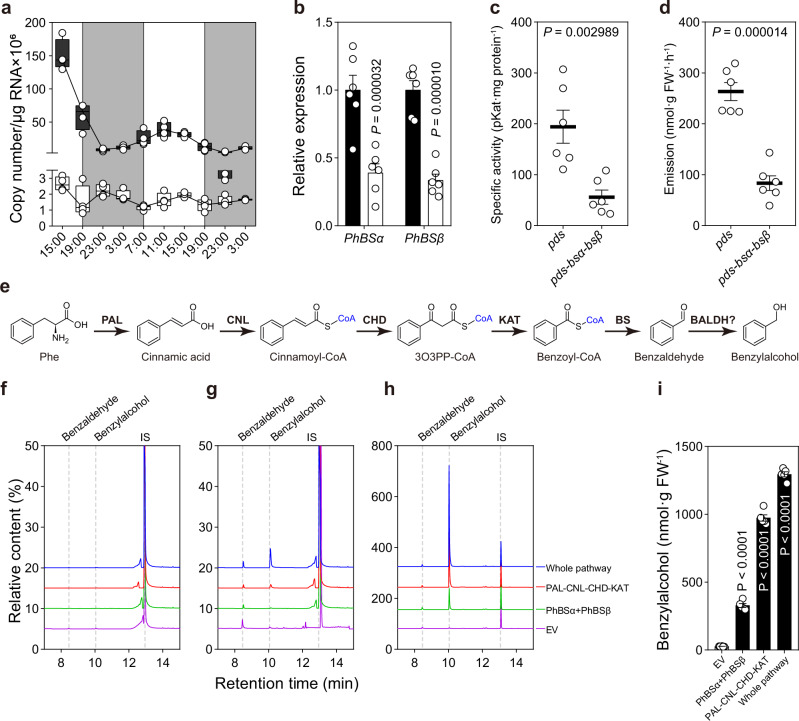
Diurnal expression and function of *PhBS* in vivo. **a** Changes in *PhBSα* (black box) and *PhBSβ* (white box) transcript levels during a normal light/dark cycle in petunia corolla harvested from day 1 post-anthesis (15:00) to day 3 post-anthesis (3:00). The *PhBSα* and *PhBSβ* expression was determined by qRT-PCR with gene-specific primers and expressed as a copy number of transcripts per microgram of total RNA × 10^6^. White and gray areas correspond to light and dark, respectively. Shown are box and whiskers plot (*n* = 4 biological replicates), center line, median; box limits, upper and lower quartiles; whiskers, minimum and maximum. **b**–**d** Effect of *PhBS* downregulation on benzaldehyde emission. **b** Transcript levels of *PhBSα* and *PhBSβ* in *pds* control (black bars) and *pds-bsα-bsβ* (white bars) in 2-day-old VIGS flowers at 21:00 h determined by qRT-PCR and presented relative to the corresponding levels in *pds* control set as 1. Data are means ± SE (*n* = 6 biological replicates). *P* values were determined by two-way ANOVA multiple comparisons test relative to the *pds* controls. **c** BS activities in crude extracts prepared from corollas of 2-day-old VIGS flowers harvested at 21:00 h. Data are means ± SE (*n* = 6 biological replicates). **d** Benzaldehyde emission in 2-day-old VIGS flowers. Volatiles were collected from 20:00 h till 21:00 h. Data are means ± SE (*n* = 6 biological replicates). *P* values shown in **c**, **d** were determined by unpaired two-tailed Student’s *t*-test relative to *pds* control. **e**–**i** Reconstitution of benzaldehyde biosynthetic pathway in *N. benthamiana* leaves. **e** Biosynthetic pathway for benzaldehyde in petunia flowers. Enzymes used for pathway reconstitution are shown in bold. The enzyme responsible for benzaldehyde reduction to benzylalcohol in petunia is unknown. **f** GC–MS analysis of infiltrated *N. benthamiana* leaves after mock feeding. **g** GC–MS analysis of infiltrated tobacco leaves after Phe feeding for 24 h. **h** GC–MS analysis of infiltrated tobacco leaves after Phe feeding and Viscozyme treatment. In **f**–**h**, constructs used for *Agrobacterium* infiltrations are shown on the right and the response of internal standard in each run was set as 100%. **i** Quantification of benzylalcohol formation in infiltrated leaves after Viscozyme treatment shown in **h**. Data are means ± SE (*n* = 5 biological replicates). *P* values were determined by ordinary one-way ANOVA multiple comparisons test relative to the EV control.

To assess the involvement of genes encoding PhBSα and PhBSβ in benzaldehyde formation, their spatial, developmental and temporal expressions were analyzed using quantitative RT-PCR (qRT-PCR) with gene-specific primers. Unexpectedly, expression of *PhBSα* was significantly higher than that of *PhBSβ* and only *PhBSα* displayed expression profiles typical for genes involved in scent production^39^. *PhBSα* was highly expressed in petal limbs and tubes, the part of the flowers that were previously shown to be primarily responsible for scent emission in petunia^40^, with low transcript levels in leaves and sepals (Supplementary Fig. 8a). *PhBSα* mRNA levels in corolla limbs were developmentally regulated increasing from buds to day 2 postanthesis and changed rhythmically during a daily light/dark cycle, peaking before 19:00 and preceding the peak of benzaldehyde emission^12,30^. In contrast, *PhBSβ* was constitutively expressed in all tissues examined. Its transcript levels in limbs moderately fluctuated during flower development (Supplementary Fig. 8b) and a daily light/dark cycle (Fig. 5a) and did not correlate with the known pattern of benzaldehyde emission^12^, suggesting that *PhBSα* determines the transcriptional specificity of *PhBS*.

### PhBS is responsible for benzaldehyde synthesis *in planta*

To investigate the in vivo function of PhBS, expressions of both *PhBSα* and *PhBSβ* were reduced in flowers by virus induced gene silencing (VIGS), a technique, which was already successfully used for studying floral scent in petunia^41–44^. A 301-bp fragment of each gene was placed in a tandem in the Tobacco rattle virus (TRV)-based vector downstream of a 300 bp fragment of *Phytoene Desaturase* (*PDS*), silencing of which was used as a visual marker for VIGS effectiveness. *PDS* silencing alone did not affect the benzaldehyde levels, which were similar to that previously detected in wild-type flowers^45^. On average, a 61% and 66% reduction were observed in *PhBSα* and *PhBSβ* mRNA levels, respectively, in flowers collected from mosaic photobleached branches of *pds*-*bsα*-*bsβ* VIGS plants relative to that in *pds* control plants (Fig. 5b). Consistent with the decrease in *PhBS* expression, benzaldehyde synthase activity in petal crude extracts and benzaldehyde emission were reduced by 71.2% and 68.3%, respectively, in *pds*-*bsα*-*bsβ* flowers relative to *pds* control (Fig. 5c and d), providing strong genetic evidence that the heterodimeric PhBS is responsible for benzaldehyde formation in petunia. In addition, emission of benzylalcohol, the product of benzaldehyde reduction, and benzylbenzoate, which relies on benzylalcohol as co-substrate, was reduced on average by 62.9% and 35.6%, respectively, in *pds*-*bsα*-*bsβ* flowers relative to control (Supplementary Fig. 9). In contrast, emission of methylbenzoate, biosynthesis of which depends on benzoic acid formed by both β-oxidative and non-β-oxidative pathways, remained unaffected. Interestingly, a 41.2% and 70.7% decrease on average was observed in phenylacetaldehyde and phenylethanol emission, respectively, while phenylethylbenzoate emission was increased by 3.07-folds (Supplementary Fig. 9). These results suggest that the reduced benzaldehyde production in the *pds*-*bsα*-*bsβ* flowers led to accumulation of benzoyl-CoA and redirection of flux towards production of phenylethylbenzoate, increasing consumption of phenylethanol (and its precursor, phenylacetaldehyde) and thus decreasing their emission. No statistically significant changes were observed in isoeugenol emission (Supplementary Fig. 9). Analysis of expression of scent biosynthetic genes by qRT-PCR revealed that their mRNA levels were unchanged in VIGS flowers relative to control (Supplementary Fig. 10).

To further evaluate the PhBS biosynthetic capacity *in planta*, both subunits were expressed in *N. benthamiana* leaves, a tissue that does not naturally produce detectable amount of benzaldehyde. In addition, the complete benzaldehyde β-oxidative biosynthetic pathway was reconstituted in *N. benthamiana* by expressing together with the *PhBS* four additional genes encoding phenylalanine ammonia lyase 1 (PhPAL1), PhCNL, PhCHD and PhKAT (Fig. 5e)^19,45,46^. Only trace amounts of benzaldehyde were detected in all three different combinations of enzymes 3 days after *N. benthamiana* leaves infiltration, which could be the result of substrate limitation (Fig. 5f). Indeed, feeding of transformed leaves with 150 mM Phe for 24 h increased not only the levels of benzaldehyde but also benzylalcohol in all groups with the highest levels in leaves expressing the whole pathway (Fig. 5g), suggesting that part of produced benzaldehyde was efficiently reduced to benzylalcohol. Notably, leaves expressing the empty vector also show an increase in benzaldehyde content, indicating the existence of some endogenous benzaldehyde biosynthetic capacity in tobacco. Indeed, a weak BS activity (<0.3 pKat mg protein^−1^) was detected in crude extracts of *N. benthamiana* leaves. Despite the low benzaldehyde levels detected, tobacco leaves with the introduced whole pathway produced 321.5 nmol g FW^−1^ more benzylalcohol than the leaves expressing the whole pathway without *PhBS* (1295 ± 19.0 versus 973.5 ± 23.4 nmol g FW^−1^ in leaves infiltrated with the whole pathway and *PAL*-*CNL*-*CHD*-*KAT*, respectively) (Fig. 5h, i). This result was consistent with the amount of benzylalcohol produced in leaves expressing only *PhBS* (327.9 ± 14.3 nmol g FW^−1^) versus the empty vector control (26.2 ± 0.7 nmol g FW^−1^) (Fig. 5h). More than 95% of detected benzylalcohol were found in its glycosylated form (Fig. 5h, i), suggesting the presence of strong glycosylation activity in tobacco leaves.

### BS homologs from other species harbor BS activity

**Fig. 6:**
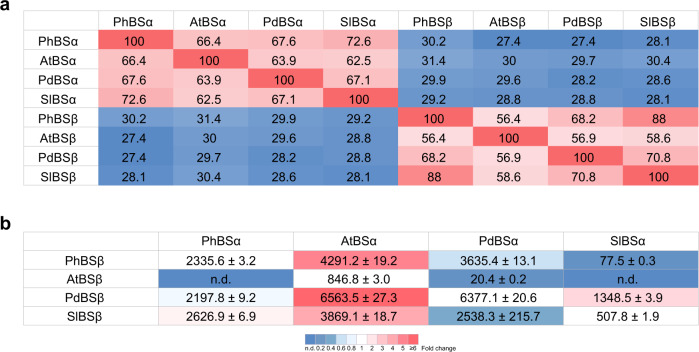
Cross-species interactions of BS subunits. **a** Protein sequence identity matrix between BS subunits from petunia (PhBS), Arabidopsis (AtBS), almond (PdBS) and tomato (SlBS). Cells are colored based on sequence identity from the lowest (blue) to the highest (red). **b** Apparent activities of cross-species BS heterodimers. Freshly purified recombinant proteins were mixed at 1:1 ratio and incubated on ice overnight before standard BS activity assays. Data are means ± SD (*n* = 3 technical replicates). Cells are colored based on fold changes of activities of each α subunit with varies β subunits relative to its original BS activity in each species.

To expand our knowledge about benzaldehyde biosynthesis in other plant species, phylogenetic analysis was performed using protein sequences of the less diverse BSβ subunits. The PhBSβ homologs were found in many land plants including monocotyledonous, dicotyledonous species and *Physcomitrella patens*, most of which have only a single copy of *BSβ* in their genomes (Supplementary Fig. 11). Three different organisms including tomato (*Solanum lycopersicum*), almond (*Prunus dulcis*) and *Arabidopsis thaliana* were chosen to test our hypothesis that a heterodimeric enzyme consisting of α and β subunits is responsible for benzaldehyde formation in other species. In tomato, benzaldehyde contributes to the fruit aroma^47^, in almonds it accumulates to high levels in leaves, flowers, and young fruits^48^, while low benzaldehyde production was found in Arabidopsis leaves^49^. Selected α subunits share 62.5–72.6% of amino acid identity, while β subunits are 56.4–88% identical with Arabidopsis β subunit being the most distantly related (Fig. 6a and Supplementary Fig. 12). Like PhBS, benzaldehyde synthases from all three organisms showed activity only when two purified recombinant subunits were combined (Supplementary Fig. 13a–d), indicating that similar mechanisms were preserved for benzaldehyde synthesis during evolutionary process. Kinetic evaluation of these enzymes revealed that petunia PhBS, almond PdBS and tomato SlBS had very similar apparent *K*_m_ values for benzoyl-CoA, which were notably higher than that of Arabidopsis AtBS (Table 3). PhBS had the highest catalytic efficiency followed by AtBS and PdBS, while SlBS had more than 5-fold lower catalytic efficiency than that of PhBS (Table 3).

To determine whether benzaldehyde synthase α subunits could interact productively with β subunits from phylogenetically distant species, enzyme assays were performed with purified recombinant petunia, Arabidopsis, almond and tomato α and β subunits in different combinations (Fig. 6b). Out of four β subunits examined, only AtBSβ subunit was not able to produce active enzymes upon interaction with PhBSα and SlBSα and formed a low activity hybrid heterodimer with PdBSα. Since the inertness of PhBSα-AtBSβ hybrid could be the result of the inability of AtBSβ to form heterodimers, purified PhBSα, PhBSβ, AtBSβ and PhBSα-AtBSβ were subjected to size exclusion chromatography. At the concentration tested (~1 mg/mL), both PhBSβ and AtBSβ exist predominantly as tetramers by themselves, while the PhBSα forms mostly multimers (Supplementary Fig. 13e). Mixing of AtBSβ with PhBSα, prevented formation of PhBSα multimers, indirectly indicating that AtBSβ indeed physically interacts with PhBSα (Supplementary Fig. 13e). The other three β subunits formed active hybrid proteins with all α subunits, but effect on activities of hybrid enzymes relative to the native benzaldehyde synthases was different. While interactions of PdBSβ and SlBSβ with PhBSα resulted in hybrid enzymes with activities almost identical to that of PhBS, interactions of the three tested β subunits with AtBSα promoted activities of hybrid proteins increasing it from 4.6- to 7.8-fold in AtBSα-SlBSβ and AtBSα-PdBSβ, respectively, relative to AtBS (Fig. 6b). In contrast, activities of hybrids with PdBSα were up to 2.5-fold lower than that of PdBS enzyme, while activities of SlBSα-PhBSβ and SlBSα-PdBSβ were 6.5-fold lower and 2.7-fold higher, respectively, when compared with SlBS. Interestingly, AtBSα-PdBSβ hybrid was as active as PdBS, but the opposite combination PdBSα-AtBSβ exhibited very low activity (Fig. 6b). Overall, our results show that BSα subunits can form active enzyme with β subunits from phylogenetically distant species, but not all BSβ subunits, at least Arabidopsis, may form active enzymes with α subunits. Moreover, the effect of BSβ subunits on activities of hybrid enzymes depends on the origin of interacting α subunit.

As the Arabidopsis genome contains *AtBS* genes, we analyzed whether the encoding heterodimeric enzyme is responsible for benzaldehyde synthesis. A search of the Arabidopsis expression datasets (ePlant, http://bar.utoronto.ca/eplant/) and proteome database (Arabidopsis PeptideAtlas, http://www.peptideatlas.org/builds/arabidopsis/) revealed that *AtBSα* and *AtBSβ* are highly expressed in flowers at both transcriptional and translational levels with relatively low expression in leaves (Supplementary Fig. 14a, d). In addition, the expression patterns of *AtBSα* and *AtBSβ* were more closely clustered with *KAT1* and *AAE12* (encoding PhCNL homolog), the core genes in the β-oxidation pathway, rather than with phenylpropanoid and lignin biosynthetic genes (Supplementary Fig. 14e), suggesting that these two genes are likely associated with the β-oxidative benzoic acid biosynthetic pathway. Metabolic profiling of Arabidopsis flowers and analysis of BS activity revealed that benzaldehyde and benzylalcohol internal pools were below detection levels while weak BS activity (0.075 pKat mg protein^−1^ on average) as well as benzaldehyde reduction activity were present in crude flower extracts. Two (CS868457 and SALK_136638C) and three (SALK_209249C, CS862843, and CS866390) T-DNA insertion lines of *AtBSα* (At3g55290) and *AtBSβ* (At3g01980), respectively, (Supplementary Fig. 15a) were obtained from ABRC and their homozygosity was confirmed by genomic PCR analysis with gene-specific primers flanking the insertion sites, which failed to amplify the respective gene regions (Supplementary Fig. 15b). All T-DNA insertions resulted in knockdown of respective genes with remaining transcript levels ranging from 9.2% to 55% relative to the wild-type plants, except for *bsα-2* mutant, which showed no changes in *AtBSα* expression (Supplementary Fig. 15c). Nevertheless, *bsα-2* mutant similar to other *bs* knockdowns had significant reduction in BS activity (Supplementary Fig. 15d, e) likely due to post-transcriptional effect of intron with T-DNA insertion on protein expression^50^ despite producing a wild-type transcript. Interestingly, downregulation of *AtBSβ* resulted in upregulation of *AtBSα*, but not vice versa (Supplementary Fig. 15c). As flower tissue contained benzaldehyde reduction activity, produced benzaldehyde by flower crude extract was rapidly converted to benzylalcohol, the levels of which were lower in all *bs* mutants relative to wild-type plants and positively correlated with benzaldehyde levels in mutants (Supplementary Fig. 15d–f). Taken together, these results suggest that heterodimeric BS is responsible for benzaldehyde biosynthesis in Arabidopsis (Supplementary Fig. 15d, e). To test whether AtBS is involved in the biosynthesis of other aromatic aldehydes, hydroxycinnamoyl-CoA thioesters were incubated with purified AtBS enzyme, and the corresponding aldehyde formation was analyzed. Similar to petunia enzyme, AtBS exhibited strict substrate specificity towards benzoyl-CoA (Supplementary Fig. 16).

## Discussion

Benzaldehyde is the most widely spread compound produced not only by plants, but also by insects, non-insect arthropods. and microbes^2^. To date, several routes for benzaldehyde formation are known in microorganisms. In *Lactobacillus plantarum*, benzaldehyde is produced from phenylalanine via phenylpyruvic acid, which is then nonenzymatically converted to aldehyde^51^. In *Pseudomonas putida*, benzaldehyde is an intermediate in the mandelic acid degradation pathway^52^, while in *Nocardia iowensis*, it is formed by a carboxylic acid reductase (CAR), a large multidomain enzyme, which first produces benzoyl-AMP from benzoic acid and ATP followed by its reduction to benzaldehyde with NADPH as a cofactor^53^. However, benzaldehyde biosynthesis in plants has been an unrevealed mystery for decades. Here, we provide biochemical (Fig. 4a and Supplementary Fig. 13b–d) and genetic evidence (Fig. 5 and Supplementary Figs. 9 and 15) that a heterodimeric enzyme composed of two distinct subunits (Fig. 4c–e, Supplementary Figs. 6 and 13b–d), both of which belong to the NAD(P)-binding Rossmann-fold superfamily, is involved in benzaldehyde formation in plants. This enzyme converts benzoyl-CoA to benzaldehyde using NADPH as the reducing power and, based on biochemical characterization of PhBS, exhibits strict substrate specificity towards benzoyl-CoA and does not accept hydroxycinnamoyl-CoA thioesters (Fig. 4b and Supplementary Fig. 16). Benzaldehyde biosynthesis occurs in peroxisomes, where both the enzyme (Supplementary Fig. 6) and its substrate, benzoyl-CoA^32^, are localized. In petunia flower peroxisomes, PhBS competes for the substrate with (i) PhTE1, a thioesterase that catalyzes the hydrolysis of benzoyl-CoA to benzoic acid, an immediate precursor of methylbenzoate, and (ii) unidentified benzoyl-CoA exporter, which exports benzoyl-CoA to the cytosol for benzylbenzoate production by BPBT^12,32^ (Supplementary Fig. 17). PhBS and PhTE1 have nearly identical affinity for benzoyl-CoA with respective apparent *K*_m_ of 677.2 ± 80.5 and 570 ± 30 μM^32^ (Table 3), but the former has almost 2-fold higher catalytic efficiency thus diverting carbon flux towards benzaldehyde production and reducing the precursor level for methylbenzoate. However, petunia flowers produce ~2.4-fold more methylbenzoate than benzaldehyde (Fig. 5d and Supplementary Fig. 9), because the latter could also be converted to benzoic acid^54^ and contribute together with the β-oxidative PhTE1-dependent pathway to methylbenzoate biosynthesis^12,33^. Moreover, reduction in *PhBS* expression and subsequent benzaldehyde formation (Fig. 5b–d) did not affect methylbenzoate emission (Supplementary Fig. 9) likely due to a redirection of the flux from benzoyl-CoA to benzoic acid. Overall, our results show that benzaldehyde is formed from benzoyl-CoA and not 3-hydroxy-3-phenylpropionyl-CoA as previously thought^12^ and explain how loss-of-function mutations in *CNL* lead to the benzaldehyde loss^5,18,24^.

In petunia flowers, benzaldehyde is one of the more abundant volatiles in the mixture of emitted compounds with relatively small internal pools, 90% of which accumulate in the cuticle relative to the rest of the cell^30^. As benzaldehyde is toxic to the cell^55^, this may explain why it was readily reduced to benzylalcohol when the complete benzaldehyde β-oxidative biosynthetic pathway was reconstituted in *N. benthamiana* (Fig. 5g–i). Analyses of *pds*-*bsα*-*bsβ* flowers, which also showed reduction in benzylalcohol emission (Supplementary Fig. 9), as well as flower protein extracts of *Atbs* mutants that produced benzylalcohol in addition to benzaldehyde in assays with benzoyl-CoA (Supplementary Fig. 15d–f), further support the existence of efficient benzaldehyde reducing capacities *in planta*.

To date, involvement of heterodimeric enzymes in metabolism has rarely been reported in plants. Examples include tryptophan synthase composed of α and β subunits^56,57^, geranyl diphosphate synthase (GPPS) containing small and large subunits^58,59^ and heterodimeric *O*-methyltransferases involved in noscapine biosynthesis in opium poppy^60,61^. However, to the best of our knowledge, benzaldehyde is the first known volatile produced by heterodimeric enzyme consisting of α and β subunits. BS are widespread in the plant kingdom. While plant genomes contain multiple copies of genes encoding α subunit homologs, most species have only a single copy of β subunit gene (Supplementary Figs. 4 and 11). Similar to other heterodimeric enzymes like *Antirrhinum majus* GPPS, where the small subunit plays a key role in regulating the formation of GPPS that produces a precursor for monoterpenes^59^, only *PhBSα* exhibits spatial, developmental and rhythmic expression, which correlates with benzaldehyde emission (Fig. 5a and Supplementary Fig. 8), and thus likely determines the transcriptional specificity of *PhBS*. In petunia flowers, *PhBSα* mRNA levels are up to 10.6-fold higher than that of *PhBSβ* (Supplementary Fig. 8a), while in Arabidopsis these genes comparably expressed (Supplementary Fig. 14). The imbalance in *PhBSα* and *PhBSβ* expression in petunia may suggest a difference in protein stability or translational efficiency since active BS requires equal molar ratio of the two subunits. Like small subunit of GPPS, which can interact with large subunits from phylogenetically distant species^62^, BSα subunits can form functional hybrid heterodimers with phylogenetically distant β subunits that generate benzaldehyde products (Fig. 6b). But not all BSβ subunits may partner with α subunits. Indeed, Arabidopsis possesses a weak AtBSβ subunit, which upon interactions with phylogenetically distant α subunits drastically reduces activity of hybrid enzymes even to undetectable level (Fig. 6b). AtBSβ is the most distantly related and shares ~57% amino acid identity with the other three analyzed β subunits (Fig. 6a). Unlike AtBSβ, AtBSα is strong and can efficiently interact with phylogenetically distant β subunits forming highly active hybrid heterodimers (Fig. 6b). While the small subunit of GPPS modifies product profile and promotes the GPPS activity of the large subunit^59,62,63^, the BSβ subunits can enhance, decrease or not alter activities of hybrid enzymes relative to the native BSs and its effect depends on the nature of interacting α subunit (Fig. 6b). Future structural analysis of BS is essential to uncover the role of each subunit in benzaldehyde synthase catalysis and to elucidate the molecular mechanism of inter-subunit interaction as well as the structural basis behind the dimer formation selectivity. Further studies are also required to assess whether (i) the occurrence of both benzaldehyde synthase subunits is universal in the plant kingdom; (ii) genes encoding both subunits are expressed in all plant species; (iii) expressed α and β subunits always form an active enzyme; (iv) all plants produce benzaldehyde or its downstream metabolite benzylalcohol and its derivatives; and (v) why the evolutionary selective pressure keeps both subunits in lineages not producing benzaldehyde, if any.

## Methods

### Plant materials

*Petunia hybrida* cv. Mitchell diploid (W115, Ball Seed Co.) plants were grown under standard greenhouse condition with a light period from 6:00 to 21:00 h. To link the simultaneous downregulation of *PhBSα* and *PhBSβ* genes via virus-induced gene silencing (VIGS) to the silencing of a marker gene^41^, previously generated pTRV2-PDS construct containing a 300 bp fragment of *Nicotiana benthamiana Phytoene Desaturase* (PDS) (CDS nucleotides 580–879) (GenBank accession no. DQ469932.1) was used. The *N. benthamiana PDS* fragment shared 94.3% sequence identity with *P. axillaris PDS* gene. Next, a 301 bp fragment of *PhBSα* (CDS nucleotides 3–303) and a 301 bp fragment of *PhBSβ* (CDS nucleotides 3–303) were tandemly cloned downstream of the *PDS* fragment in pTRV2-PDS plasmid using ClonExpress II One Step Cloning Kit (Vazyme Biotech Co., Piscataway, NJ, USA), yielding the final construct pTRV2-PDS-PhBSαβ. Before cloning, all fragments were verified to target only the desired genes and not to lead to off-target interference using the Sol Genomics Network VIGS Tool (http://vigs.solgenomics.net/). The analysis was performed by comparing the *PDS*, *PhBSα* and *PhBSβ* VIGS target sequences against the *P. axillaris* and *P. inflata* (parental lines of *P. hybrida*) genomes^64^ using default parameters. This analysis revealed that no other genes would be targeted by the *PDS*, *PhBSα* and *PhBSβ* double-stranded RNAs generated during the replication of viral genomes. *PhBSα* and *PhBSβ* fragments were PCR amplified from *P. hybrida* petal cDNA using gene-specific primers (Supplementary Table 1). All binary vectors, including pTRV1 (GenBank accession no. AF406990), pTRV2-PDS and pTRV2-PDS-PhBSαβ, were transformed into *Agrobacterium tumefaciens* strain GV3101. Single colonies for each construct were cultured at 28 °C in LB medium containing 50 mg/L rifampicin, 50 mg/L gentamycin and 50 mg/L kanamycin to OD_600_ of 2.4. Cells were pelleted, washed with infiltration buffer (50 mM MES, pH 5.7, 2 mM Na_3_PO_4_, 0.5% glucose and 200 μM acetosyringone), and incubated in the same buffer for 2 h at room temperature^65^. Before infiltration, cultures containing pTRV1 and pTRV2 were mixed at 1:1 ratio to reach a final OD_600_ of 2.0. Using a blunt end syringe, the suspension was injected into the abaxial surface of all leaflets of 3- to 4-week-old petunia seedlings. Infiltrated seedlings were grown under normal greenhouse conditions until plant blooming. Plants infiltrated with pTRV1 and pTRV2-PDS were served as negative controls.

The Arabidopsis T-DNA insertion lines are obtained from Arabidopsis Biological Resource Center (ABRC, Ohio State University, USA, https://abrc.osu.edu) and propagated under normal growth room conditions. Genomic DNA was extracted from several individuals of each line. Homozygous individuals were identified using primers (Supplementary Table 1) designed with SIGnAL (Salk Institute Genomic Analysis Laboratory) online T-DNA Primer Design program (http://signal.salk.edu/tdnaprimers.2.html).

### Labeling experiments

Feeding experiments were performed largely according to previous publication^12^ with minor modifications. Deuterium labeled l-phenylalanine (^2^H_8_, 98%) and benzoic acid (ring-^2^H_5_, 98%) were purchased from Cambridge Isotope Laboratories (Andover, MA, USA). 50 mM [^2^H_8_]-Phe (prepared in 5% sucrose solution) or 100 mM [^2^H_5_]-benzoic acid (in 5% sucrose solution, neutralized with sodium hydroxide) were fed to excised limbs of 2-day-old petunia flowers for 2 h, following by scent collection for 4 h from 18:00 to 22:00 h. Emitted VOCs were analyzed with GC-MS as described in VOC profiling section.

### Partial purification of benzaldehyde synthase

Petunia flower limbs were collected from 9 p.m. to 10 p.m., flash frozen in liquid nitrogen and stored at −80 °C until protein purification. All purification procedures were performed on ice or at 4 °C except as noted. In a typical purification, 60 g of petal tissue were ground in liquid nitrogen to a fine powder using a mortar and pestle. 240 mL of protein extraction buffer A containing 100 mM Tris, pH 7.4, 150 mM NaCl, 1 mM ethylenediaminetetraacetic acid, 1% (v/v) Triton X-100, 10% (v/v) glycerol, 10 mM dithiothreitol, and 1 mM phenylmethanesulfonyl fluoride were immediately added to the powder. The slurry was ground for additional 10 min with a pestle and incubated on ice for 30 min with frequent mixing. After centrifugation at 10,000×*g* for 30 min, the supernatant was passed through double layer of Miracloth (Calbiochem) and ammonium sulfate was added sequentially in a 10% increment starting from 40% saturation up to 80% saturation. After 20 min of gentle rotation at each step, ammonium sulfate precipitation solution was centrifuged at 12,000×*g* for 10 min. Protein pellets from each precipitation step were re-suspended in 24 mL of buffer B (20 mM Tris, pH 7.4, 10% (v/v) glycerol) and tested for the BS activity. The fraction of 50–60% ammonium sulfate saturation was used for further purification. The fraction was diluted to 144 mL with buffer B, passed through a 0.45 μm filter and loaded onto a DEAE-cellulose anion exchange column (25 × 65 mm column containing 10 g DE53, Whatman) at the flow rate of 2 mL min^−1^ using the FPLC system (AKTA, GE Healthcare). After washing of the unabsorbed material with 60 mL of buffer B, proteins were eluted from the column with a linear gradient (300 mL) from 0 to 500 mM NaCl in buffer B. Fractions of 5 mL were collected and assayed for BS activity. Fractions 14 and 15 with the highest BS activity eluted at about 125 mM NaCl were pooled, diluted to 15 mL with buffer B, and loaded at the flow rate of 0.5 mL min^−1^ onto anion exchange column Mono Q 5/50 GL (GE Healthcare) pre-equilibrated with buffer B. Column was washed with 5 mL of buffer B and the bound protein was eluted using a linear gradient (20 mL) from 0 to 500 mM NaCl in buffer B. Fractions of 0.5 mL were collected and analyzed for BS activity. Fraction 24 with the highest BS activity eluted at about 170 mM NaCl was further subjected to size-exclusion gel filtration chromatography on a Superose 12 10/300 GL column (GE Healthcare) using buffer C (20 mM Tris, pH 7.4, 150 mL NaCl) for column equilibration and elution at the flow rate of 0.4 mL min^−1^. Fractions of 0.5 mL were collected and tested for BS activity.

Fractions from all purification steps were analyzed with 12% SDS–PAGE followed by gel staining with Coomassie brilliant blue (CBB) R-250. Three fractions from Mono Q chromatography (MonoQ 24 to 26) and one fraction from gel filtration chromatography (SEC 24) containing BS activity were subjected to proteomic analysis at Purdue Proteomics Facility (Bindley Bioscience Center, Purdue University) as described previously^66^. The MS/MS spectra were searched against the *P. axillaris* protein sequences downloaded from Sol Genomics Network draft genome sequence v1.6.2 (https://solgenomics.net/organism/Petunia_axillaris/genome) and the abundance of detected proteins is shown in Supplementary Data 1.

### Molecular weight determination

To determine the apparent molecular weight of native PhBS, the size-exclusion chromatography Superose 12 10/300 GL column was calibrated with following markers from Gel Filtration Markers Kit (Millipore Sigma): cytochrome *c* (12.4 kDa), carbonic anhydrase (29 kDa), bovine serum albumin (66 kDa), alcohol dehydrogenase (150 kDa), and β-amylase (200 kDa). To determine the apparent molecular weight of purified recombinant BS proteins, Superdex 200 Increase 10/300 GL size-exclusion column (GE Healthcare) was used with phosphate-buffered saline (PBS) for column equilibration and elution. The column was calibrated with following markers: bovine thyroglobulin (670 kDa), bovine gamma globulin (158 kDa), chicken ovalbumin (44 kDa), horse myoglobulin (17 kDa), and vitamin B-12 (1.4 kDa).

### Heterologous expression and purification of recombinant proteins

Recombinant BS proteins were produced in *Escherichia coli* by expressing the corresponding coding regions (CDS) subcloned into an expression vector pMAL-c5X (New England BioLabs, NEB) containing a Maltose Binding Protein (MBP) tag using ClonExpress II One Step Cloning Kit (Vazyme). Petunia and Arabidopsis CDSs were PCR amplified from the corresponding flower cDNAs with gene-specific primers (Supplementary Table 1), while almond (PdBSα: GenBank accession no. XP_034223352.1; PdBSβ: GenBank accession no. XP_034208025.1) and tomato proteins (SlBSα: GenBank accession no. XP_004249319.1; SlBSβ: GenBank accession no. XP_004243555.1) were identified by BLAST of amino acid sequences of PhBS subunits against NCBI non-redundant protein database. Candidates with the highest sequence identities were chosen, and their corresponding CDSs were codon-optimized and synthesized (GenScript, Piscataway, NJ, USA). After sequence verification, the desired plasmids were transformed into Rosetta2 (DE3) pLysS competent cells (EMD Millipore, Billerica, MA, USA). Single colonies were picked and cultured overnight in 2 ml LB medium containing 100 mg/L carbenicillin and 34 mg/L chloramphenicol at 37 °C. Overnight cultures were then diluted 100-fold with LB medium and continued to culture at 37 °C until OD_600_ reached ~0.5. After cooling the cell culture on ice for 10 min, isopropyl β-d-1-thiogalactopyranoside (IPTG) was added to a final concentration of 0.1 mM and cells were incubated at 28 °C with shaking for additional 2.5~3 h. After harvesting cells by centrifugation, their lysis and protein purification were performed according to the manufacturer’s protocol of Amylose Resin (NEB).

For pull-down experiments, the PhBSβ CDS was amplified, cloned into the *Nde*I and *Hind*III sites of pET32b expression vector in-frame with a C-terminal 6×His-tag (Novagen, Madison, WI, USA) using ClonExpress II One Step Cloning Kit (Vazyme), and the protein was expressed in *E. coli*. Next, the MBP tag (~42.5 kDa) was removed from the purified MBP-PhBSα by digestion with Factor Xa protease (NEB) overnight at 4 °C, and PhBSα was mixed with bacterial cell lysate containing soluble PhBSβ-His_6_. The reconstituted PhBS complex was purified using nickel-nitrilotriacetic acid (Ni-NTA) agarose (Qiagen, Hilden, Germany) following the manufacturer’s protocol and analyzed for BS activity. Alternatively, bacterial lysates containing MBP-PhBSα were mixed with PhBSβ-His_6_ and the reconstituted PhBS complex was purified using amylose resin. Co-purified proteins were analyzed with 15% SDS-PAGE followed by the CBB gel staining.

### Benzaldehyde synthase activity assay

Benzaldehyde synthase activity assays were carried out in 100 μL reaction mixture containing 50 mM Bis–Tris buffer, pH 6.5, 200 μM benzoyl-CoA, and 2 mM freshly prepared NADPH. The reaction was initiated by adding a protein and incubated at 28 °C for 30 min. For benzoyl-CoA *K*_m_, various benzoyl-CoA concentrations ranging from 56.25 μM to 3.6 mM were used in the presence of 4 mM NADPH with the following amounts of recombinant proteins purified with amylose resin after mixing α and β subunits at 1:1 molar ratio: 1.3 μg (petunia PhBS), 1.7 μg (Arabidopsis AtBS), 2.0 μg (almond PdBS), and 2.0 μg (tomato SlBS). For NADPH *K*_m_, NADPH concentrations ranged from 62.5 μM to 4 mM in the presence of 3.6 mM benzoyl-CoA and 1.3 μg of purified MBP-tagged PhBS containing both subunits at 1:1 molar ratio. The reactions were terminated by adding 100 μL of 100% ice-cold MeOH. After centrifugation at 15,000×*g* for 20 min, 5 μL of the supernatant were subjected to high-performance liquid chromatography (HPLC) analysis using an Agilent 1260 Infinity II system (Santa Clara, CA) equipped with an InfinityLab Poroshell 120 EC-C18 column (3.0 × 150 mm, 2.7 μm) maintained at 35 °C. Products were separated with a 15-min linear gradient of 10% aqueous acetonitrile solution to 100% acetonitrile and monitored at 248 nm using a diode array detector (DAD). Benzaldehyde was identified by comparing the retention time and absorption spectrum with authentic standard. Quantitation was achieved by using standard curve generated from authentic standard.

Product verification was performed by gas chromatography-mass spectrometry (GC-MS). Crude protein extracts were obtained by extraction of petunia petal tissue with buffer A (3:1 [v/w] buffer/tissue). After centrifugation of slurry at 15,000×*g* for 20 min, the supernatant was desalted with Econo-Pac 10DG Columns (Bio-Rad, Hercules, CA) according to the manufacturer’s protocol and 20 μL of desalted protein was used in BS activity assays. The reaction was performed for 30 min at 28 °C and the product was extracted with 200 μL dichloromethane (DCM) containing 1 μg naphthalene (internal standard). After centrifugation at 15,000×*g* for 20 min, 2 μL of the bottom DCM phase was injected in GC-MS.

For analysis of BS activity in Arabidopsis flowers, newly bloomed flowers and unopened flower buds from wild type ecotype Col-0 and mutants were flash frozen in liquid nitrogen, ground into fine powder and proteins were extracted with buffer A (3:1 [v/w] buffer/tissue). After centrifugation, protein concentration in the supernatant was quantified using Bradford reagent (Bio-Rad). 100 μg of total protein was used in activity assays in the presence of 500 μM benzoyl-CoA and 2 mM NADPH. Reactions were incubated at 28 °C for 2 h before extraction with DCM and quantitation using GC–MS as described above.

All enzyme assays were performed at an appropriate enzyme concentration so that reaction velocity was proportional to enzyme concentration and linear during the incubation time period. *K*_m_ and V_max_ were determined by non-linear fit to the Michaelis–Menten equation using GraphPad Prism, v8.0.0. Triplicate assays were performed for all data points.

### Yeast two-hybrid assay

Coding sequences of both *PhBSα* and *PhBSβ* were PCR amplified using gene-specific primers (Supplementary Table 1) and cloned into *Eco*RI sites of the GAL4 yeast two-hybrid (Y2H) system plasmids pGADT7-AD and pGBKT7-BD, containing the activation domain (AD) and the DNA-binding domain (BD), respectively, using ClonExpress II One Step Cloning Kit (Vazyme). Different combinations of AD and BD plasmids were co-transformed into yeast strain Y2H Gold and selected on -*leu*/-*trp* double drop out (DDO) medium. After incubation at 30 °C for 3 days, individual large colonies (>2 mm diameter) survived on DDO plates were resuspended in 100 μL sterile TE buffer (10 mM Tris, 1 mM EDTA, pH7.5), subjected to a series of stepwise dilutions and blotted onto -*leu*/-*trp*/-*his* triple drop out (TDO) medium to test their growth response at 30 °C. All TDO plates were image documented for colony growth 48 h after blotting to control false positive growth, which is common after prolonged incubation. Constructs containing PhBS subunits were also co-transformed with empty vectors to exclude unspecific interactions between PhBS subunits and AD/BD domains. pGADT7-T/pGBKT7-53 and pGADT7-T/pGBKT7-lam were used as positive and negative controls of the GAL4 Y2H system.

### Pathway reconstitution in *Nicotiana benthamiana*

The coding sequences of *PhPAL1*, *PhCNL*, *PhCHD*, *PhKAT*, *PhBSα* and *PhBSβ* were PCR amplified with gene-specific primers (Supplementary Table 1) from petunia flower cDNAs and cloned into binary vector pCNHP using ClonExpress II One Step Cloning Kit (Vazyme). After sequence verification, plasmids were transformed into *Agrobacterium tumefaciens* strain GV3101. Single colonies for each construct were picked and cultured at 28 °C in 3 mL of LB medium supplied with 50 mg/L rifampicin, 50 mg/L gentamycin and 50 mg/L kanamycin to OD_600_ of about 2.0. Bacterial cultures were pelleted, washed with 10 mM MgCl_2_ solution containing 200 μM acetosyringone, and incubated in the same solution for additional 2 h at room temperature. Before infiltration, cultures were mixed to reach a final OD_600_ of 1.0 for each of the constructs used. The suspension was injected into the abaxial surface of 4–6-week-old *N. benthamiana* leaves with a needleless syringe. Infiltrated plants were grown under dim light for 3 days before leaves were detached and submerged in 150 mM Phe solution for 24 h. Leaf tissues were then ground into fine powder in liquid nitrogen. 200 mg of ground tissue were extracted twice with 500 μL of DCM containing 1 μg naphthalene (internal standard), concentrated to about 200 μL and subjected to GC–MS analysis. To release benzylalcohol from its potential glucosides, 200 mg of ground tissue was suspended in 500 μL phosphate-citrate buffer (150 mM, pH 5.0) and 100 μL Viscozyme L (Sigma-Aldrich) was added^67^. Samples were incubated at 37 °C for 6 h with frequent mixing. Metabolites were extracted twice with DCM and subjected to GC–MS analysis as described above.

### Subcellular localization and BiFC

To examine the subcellular localization of PhBS subunits, full-length CDS of each subunit was PCR amplified using gene-specific primers (Supplementary Table 1) and cloned into binary plasmids pK7WGF2 and pCNHP-EYFP, which express fusion proteins with N-terminal green fluorescent protein (GFP) and C-terminal enhanced yellow fluorescent protein (EYFP), respectively. For BiFC, *PhBSα* and *PhBSβ* CDSs were amplified and cloned into plasmids pCNHP-nEYFP-C and pCNHP-cEYFP-C, which express fusion proteins with either N-terminal half of EYFP (nEYFP) or C-terminal half of EYFP (cEYFP) at their N-terminus, respectively. The resulting constructs were used for transient expression in *N. benthamiana* leaves. Transformation of constructs into *A. tumefaciens* and infiltration of cell cultures in tobacco leaves were performed as described above except that the final OD_600_ of the culture was adjusted to 0.6. Plasmid expressing mCherry-labeled peroxisomal marker obtained from ABRC (ABRC stock: CD3-984) was co-infiltrated with PhBS subunit expression constructs. 48 h after infiltration, the fluorescent signals in leaves were imaged using a Zeiss LSM-880 laser-scanning confocal microscope (Zeiss, Thornwood, NY, USA). The excitation wavelength and emission bandwidth recorded for each fluorescent protein as well as chlorophyll autofluorescence were optimized by the default presets in the ZEN 2.6 software (Zeiss) and were as follows: EYFP (excitation 514 nm, emission 519–583 nm), GFP (excitation 488 nm, emission 493–556 nm), mCherry (excitation 561 nm, emission 580–651 nm), chlorophyll autofluorescence (excitation 633 nm, emission 652–721 nm).

### Analysis of floral volatiles

Petunia flower volatiles were collected by a closed-loop stripping method and analyzed by GC-MS as described previously^66^ with minor modifications. Briefly, volatiles were collected from 20:00 to 21:00 h from a minimum of three 2-day-old flowers per biological replicate. Absorbed volatiles were eluted from collection traps containing 20 mg of Porapak Q (80-100 mesh) (Waters, Milford, MA) with 200 μl DCM supplemented with 1 μg of naphthalene as internal standard. For the analysis of internal pools, 100 mg of ground tissue was extracted twice at 4 °C with 500 μL DCM containing 1 μg naphthalene as internal standard. The extracts were pooled together and concentrated to about 200 μL under a mild stream of nitrogen gas. Samples were analyzed on an Agilent 7890B-5977B GC-MS system using an analytical method translated from established method with Agilent 6890N-5975B GC-MS system as previously described^66^. Quantitation of different volatile compounds was performed based on standard curves generated with authentic standards.

### RNA extraction, reverse transcription, and qRT-PCR

Sample collection, RNA isolation and quantitative real-time PCR (qRT-PCR) were performed as described previously^19^. Briefly, samples were collected from tissues and time points indicated in the text and RNA was extracted using the Spectrum Plant Total RNA Kit (Millipore sigma). About 1 μg of total RNA was reverse transcribed to first strand cDNA in 10 μl reaction using the EasyScript cDNA synthesis kit (Applied Biological Materials Inc.). Individual qRT-PCR reactions were performed in 5 μL of Fast SYBR Green Master Mix (Applied Biosystems) with gene-specific primers shown in Supplementary Table 1 using a StepOne Real-Time PCR System (Applied Biosystems). For relative expression quantification, *Elongation factor 1-alpha* (*PhEF1α*) and *Actin 2* (*AtACT2*, AT3G18780) were used as internal reference genes for petunia and Arabidopsis cDNAs, respectively. Absolute quantities of transcripts were calculated based on standard curves generated from purified templates of the corresponding CDSs and expressed as copy numbers per microgram of total RNA.

### Sequence alignment and phylogenetic analysis

PhBSβ homologs in land plants were obtained by EggNOG^68^ Sequence search (http://eggnog5.embl.de/dev/#/app/seqscan) using PhBSβ amino acid sequence. Homologs of both PhBSα and PhBSβ from *Petunia* genus were obtained by BLASTP search against published genomes on Solanaceae Genome Project website (https://solgenomics.net/). The amino acid sequences were aligned using MUSCLE algorithm in MEGA7 package^69^. The Neighbor-Joining algorithm in MEGA7 program was used to build the phylogenetic tree shown on Supplementary Fig. 4. Maximum-likelihood phylogenetic tree was constructed using MEGA7 with the suitable model inferred by “Find Best DNA/Protein Models (ML)” algorithm. Phylogenetic trees were tested with Bootstrap method for 500 replications (Maximum-likelihood trees) or 1000 replications (Neighbor-Joining tree).

### In silico analysis of Arabidopsis genes

Tissue specific and developmental expression data of *AtBSα* and *AtBSβ* were obtained from ePlant^70^ website (https://bar.utoronto.ca/eplant/). Data for peptide enrichment of AtBSα and AtBSβ proteins in different tissues were retrieved from Arabidopsis PeptideAtlas^71^ project website (http://www.peptideatlas.org/builds/arabidopsis/). Hierarchical clustering plot of *AtBS* genes as well as genes involved in β-oxidation and lignin biosynthesis pathways was generated using ATTED-II^72^ Hcluster tool (https://atted.jp/top_draw/#Hcluster).

### Reporting summary

Further information on research design is available in the Nature Research Reporting Summary linked to this article.

## Supplementary information

Supplementary Information

Description of Additional Supplementary Files

Supplementary Data 1

Supplementary Data 2

Reporting Summary

## Data Availability

The data supporting the findings of this study are available within the article and its Supplementary Information files. The sequences reported in this paper have been deposited in GenBank database with the following accession numbers OK095279 for PhBSα, OK095280 for PhBSβ, OK095281 for AtBSα, OK095282 for AtBSβ, OK095283 for PdBSα, OK095284 for PdBSβ, OK095285 for SlBSα and OK095286 for SlBSβ. Proteomic raw data and MaxQuant search results have been made publicly available through MassIVE (https://massive.ucsd.edu/) with submission ID: MSV000088175. Source data are provided with this paper.

## Source data

Source Data
