# Supplementary material for: A peroxisomal heterodimeric enzyme is involved in benzaldehyde synthesis in plants
Source: Nat Commun. 2022 Mar 15;13:1352. doi: 10.1038/s41467-022-28978-2 (PMC8924275; doi:10.1038/s41467-022-28978-2)
Supplement: Supplementary file 1 — Supplementary Information [file 41467_2022_28978_MOESM1_ESM.pdf]

A peroxisomal heterodimeric enzyme is involved in benzaldehyde synthesis in plants

Xing-Qi Huang *et al.*

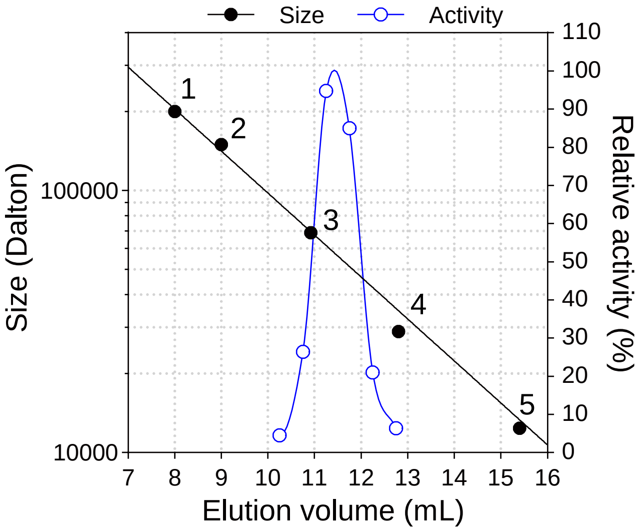

Supplementary Figure 1 | Determination of apparent molecular weight of native PhBS. A small fraction (200 µL) of crude petunia flower protein extract was loaded onto a Superose 12 10/300 size exclusion column and eluted with buffer C (see Methods for buffer composition). Fractions of 0.5 mL were assayed for benzaldehyde synthase activity, which was plotted against elution volume (blue line). The column was calibrated with the following standard proteins: (1) β-amylase (200 kDa), (2) alcohol dehydrogenase (150 kDa), (3) bovine serum albumin (66 kDa), (4) carbonic anhydrase (29 kDa), and (5) cytochrome *c* (12.4 kDa) and their elution behavior is shown with black line.

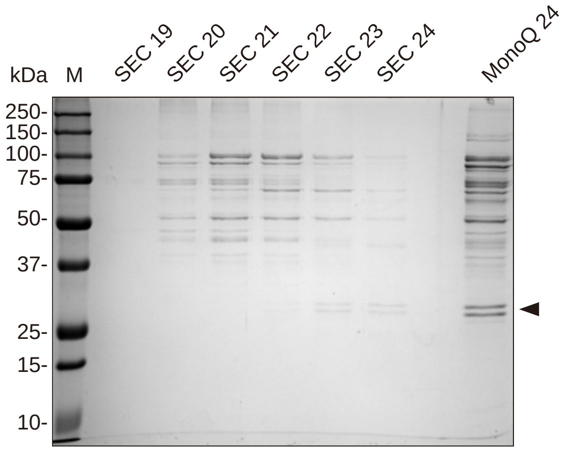

Total activity	22	96	157
Intensity density (upper band)	0.007	0.052	0.056
Intensity density (lower band)	0.013	0.061	0.066

Supplementary Figure 2 | SDS-PAGE analysis of fractions from size exclusion chromatography. SEC 19 to SEC 24 are fractions from size exclusion chromatography on a Superose 12 10/300 column of MonoQ fraction 24 (10 µL of which are loaded in the right lane). Fractions of 0.5 mL were collected from size exclusion chromatography, 20 µL were used for analysis of BS activity while the rest was precipitated with acetone, re-dissolved in 10 µL of loading buffer and run on 12% SDS-PAGE. Total BS activity (pKat·mg protein^{-1}) and intensity density of BS subunits for fractions SEC 22 to SEC 24 are shown below the gel. M, protein molecular weight standards. Triangle indicates the position of BS subunits. The experiment was repeated three times with similar results.

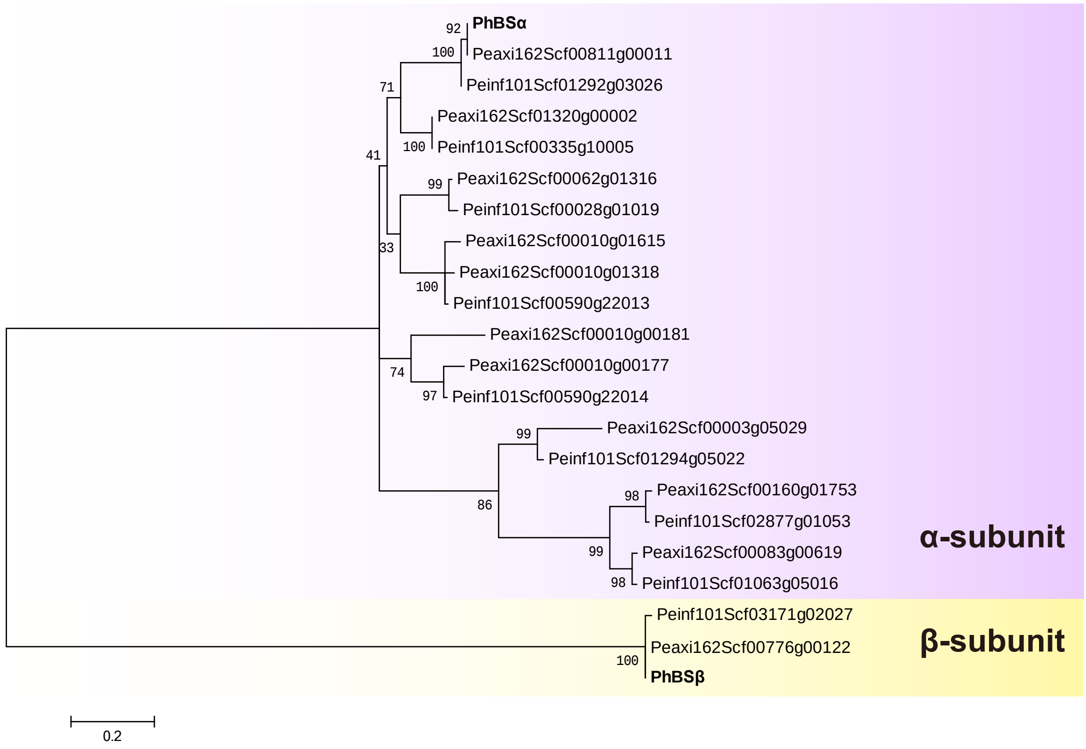

Supplementary Figure 3 | Phylogenetic analysis of benzaldehyde synthase homologs in *Petunia* genus. The evolutionary history was inferred by using the Maximum Likelihood method based on the JTT matrix-based model. The tree with the highest log likelihood (-1988.16) is shown. The percentage of trees in which the associated taxa clustered together is shown next to the branches. Initial tree(s) for the heuristic search were obtained automatically by applying Neighbor-Join and BioNJ algorithms to a matrix of pairwise distances estimated using a JTT model, and then selecting the topology with superior log likelihood value. A discrete Gamma distribution was used to model evolutionary rate differences among sites (8 categories (+G, parameter = 1.3343)). The rate variation model allowed for some sites to be evolutionarily invariable ([+I], 8.33% sites). The tree is drawn to scale, with branch lengths measured in the number of substitutions per site.

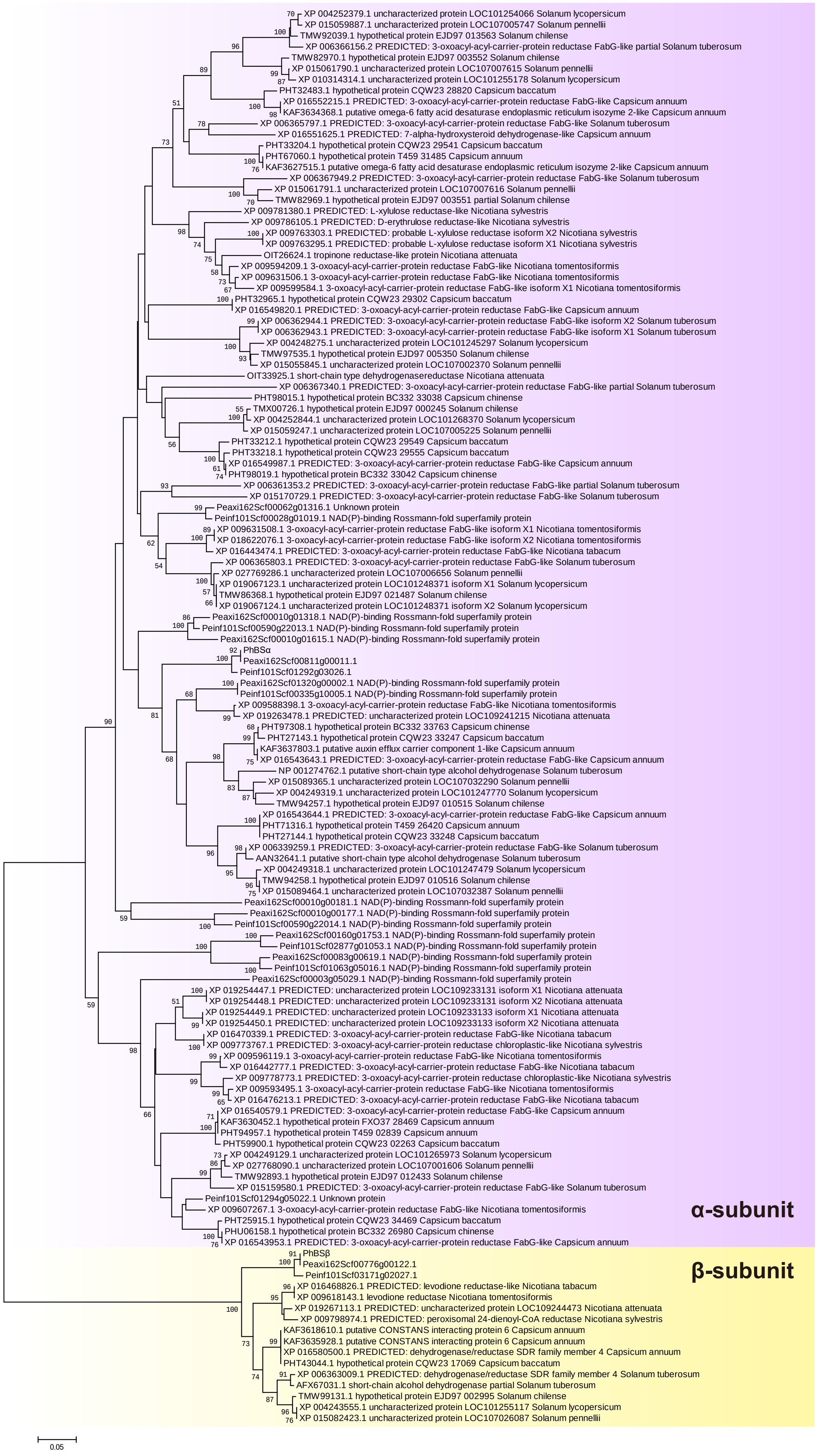

XP 004252379.1 uncharacterized protein LOC101254066 Solanum lycopersicum
XP 015059887.1 uncharacterized protein LOC107005747 Solanum pennellii
TMW92039.1 hypothetical protein EJD97 013563 Solanum chilense
XP 006366156.2 PREDICTED: 3-oxoacyl-acyl-carrier-protein reductase FabG-like partial Solanum tuberosum
TMW82970.1 hypothetical protein EJD97 003552 Solanum chilense
XP 015061790.1 uncharacterized protein LOC107007615 Solanum pennellii
XP 010314314.1 uncharacterized protein LOC101255178 Solanum lycopersicum
PHT32483.1 hypothetical protein CQW23 28820 Capsicum baccatum
XP 016552215.1 PREDICTED: 3-oxoacyl-acyl-carrier-protein reductase FabG-like Capsicum annuum
KAF3634368.1 putative omega-6 fatty acid desaturase endoplasmic reticulum isozyme 2-like Capsicum annuum
XP 006365797.1 PREDICTED: 3-oxoacyl-acyl-carrier-protein reductase FabG-like Solanum tuberosum
XP 016551625.1 PREDICTED: 7-alpha-hydroxysteroid dehydrogenase-like Capsicum annuum
PHT33204.1 hypothetical protein CQW23 29541 Capsicum baccatum
PHT67060.1 hypothetical protein T459 31485 Capsicum annuum
KAF3627515.1 putative omega-6 fatty acid desaturase endoplasmic reticulum isozyme 2-like Capsicum annuum
XP 006367949.2 PREDICTED: 3-oxoacyl-acyl-carrier-protein reductase FabG-like Solanum tuberosum
XP 015061791.1 uncharacterized protein LOC107007616 Solanum pennellii
TMW82969.1 hypothetical protein EJD97 003551 partial Solanum chilense
XP 009781380.1 PREDICTED: L-xylulose reductase-like Nicotiana sylvestris
XP 009786105.1 PREDICTED: D-erythrulose reductase-like Nicotiana sylvestris
XP 009763303.1 PREDICTED: probable L-xylulose reductase isoform X2 Nicotiana sylvestris
XP 009763295.1 PREDICTED: probable L-xylulose reductase isoform X1 Nicotiana sylvestris
OIT26624.1 tropinone reductase-like protein Nicotiana attenuata
XP 009594209.1 3-oxoacyl-acyl-carrier-protein reductase FabG-like Nicotiana tomentosiformis
XP 009631506.1 3-oxoacyl-acyl-carrier-protein reductase FabG-like Nicotiana tomentosiformis
XP 009599584.1 3-oxoacyl-acyl-carrier-protein reductase FabG-like isoform X1 Nicotiana tomentosiformis
PHT32965.1 hypothetical protein CQW23 29302 Capsicum baccatum
XP 016549820.1 PREDICTED: 3-oxoacyl-acyl-carrier-protein reductase FabG-like Capsicum annuum
XP 006362944.1 PREDICTED: 3-oxoacyl-acyl-carrier-protein reductase FabG-like isoform X2 Solanum tuberosum
XP 006362943.1 PREDICTED: 3-oxoacyl-acyl-carrier-protein reductase FabG-like isoform X1 Solanum tuberosum
XP 004248275.1 uncharacterized protein LOC101245297 Solanum lycopersicum
TMW97535.1 hypothetical protein EJD97 005350 Solanum chilense
XP 015055845.1 uncharacterized protein LOC107002370 Solanum pennellii
OIT33925.1 short-chain type dehydrogenasereductase Nicotiana attenuata
XP 006367340.1 PREDICTED: 3-oxoacyl-acyl-carrier-protein reductase FabG-like partial Solanum tuberosum
PHT98015.1 hypothetical protein BC332 33038 Capsicum chinense
TMX00726.1 hypothetical protein EJD97 000245 Solanum chilense
XP 004252844.1 uncharacterized protein LOC101268370 Solanum lycopersicum
XP 015059247.1 uncharacterized protein LOC107005225 Solanum pennellii
PHT33212.1 hypothetical protein CQW23 29549 Capsicum baccatum
PHT33218.1 hypothetical protein CQW23 29555 Capsicum baccatum
XP 016549987.1 PREDICTED: 3-oxoacyl-acyl-carrier-protein reductase FabG-like Capsicum annuum
PHT98019.1 hypothetical protein BC332 33042 Capsicum chinense
XP 006361353.2 PREDICTED: 3-oxoacyl-acyl-carrier-protein reductase FabG-like partial Solanum tuberosum
XP 015170729.1 PREDICTED: 3-oxoacyl-acyl-carrier-protein reductase FabG-like Solanum tuberosum
Peaxi162Scf00062g01316.1 Unknown protein
Peinf101Scf00028g01019.1 NAD(P)-binding Rossmann-fold superfamily protein
XP 009631508.1 3-oxoacyl-acyl-carrier-protein reductase FabG-like isoform X1 Nicotiana tomentosiformis
XP 018622076.1 3-oxoacyl-acyl-carrier-protein reductase FabG-like isoform X2 Nicotiana tomentosiformis
XP 016443474.1 PREDICTED: 3-oxoacyl-acyl-carrier-protein reductase FabG-like Nicotiana tabacum
XP 006365803.1 PREDICTED: 3-oxoacyl-acyl-carrier-protein reductase FabG-like Solanum tuberosum
XP 027769286.1 uncharacterized protein LOC107006656 Solanum pennellii
XP 019067123.1 uncharacterized protein LOC101248371 isoform X1 Solanum lycopersicum
TMW86368.1 hypothetical protein EJD97 021487 Solanum chilense
XP 019067124.1 uncharacterized protein LOC101248371 isoform X2 Solanum lycopersicum
Peaxi162Scf00010g01318.1 NAD(P)-binding Rossmann-fold superfamily protein
Peinf101Scf00590g22013.1 NAD(P)-binding Rossmann-fold superfamily protein
Peaxi162Scf00010g01615.1 NAD(P)-binding Rossmann-fold superfamily protein
PhBSα
Peaxi162Scf00811g00011.1
Peinf101Scf01292g03026.1
Peaxi162Scf01320g00002.1 NAD(P)-binding Rossmann-fold superfamily protein
Peinf101Scf00335g10005.1 NAD(P)-binding Rossmann-fold superfamily protein
XP 009588398.1 3-oxoacyl-acyl-carrier-protein reductase FabG-like Nicotiana tomentosiformis
XP 019263478.1 PREDICTED: uncharacterized protein LOC109241215 Nicotiana attenuata
PHT97308.1 hypothetical protein BC332 33763 Capsicum chinense
PHT27143.1 hypothetical protein CQW23 33247 Capsicum baccatum
KAF3637803.1 putative auxin efflux carrier component 1-like Capsicum annuum
XP 016543643.1 PREDICTED: 3-oxoacyl-acyl-carrier-protein reductase FabG-like Capsicum annuum
NP 001274762.1 putative short-chain type alcohol dehydrogenase Solanum tuberosum
XP 015089365.1 uncharacterized protein LOC107032290 Solanum pennellii
XP 004249319.1 uncharacterized protein LOC101247770 Solanum lycopersicum
TMW94257.1 hypothetical protein EJD97 010515 Solanum chilense
XP 016543644.1 PREDICTED: 3-oxoacyl-acyl-carrier-protein reductase FabG-like Capsicum annuum
PHT71316.1 hypothetical protein T459 26420 Capsicum annuum
PHT27144.1 hypothetical protein CQW23 33248 Capsicum baccatum
XP 006339259.1 PREDICTED: 3-oxoacyl-acyl-carrier-protein reductase FabG-like Solanum tuberosum
AAN32641.1 putative short-chain type alcohol dehydrogenase Solanum tuberosum
XP 004249318.1 uncharacterized protein LOC101247479 Solanum lycopersicum
TMW94258.1 hypothetical protein EJD97 010516 Solanum chilense
XP 015089464.1 uncharacterized protein LOC107032387 Solanum pennellii
Peaxi162Scf00010g00181.1 NAD(P)-binding Rossmann-fold superfamily protein
Peaxi162Scf00010g00177.1 NAD(P)-binding Rossmann-fold superfamily protein
Peinf101Scf00590g22014.1 NAD(P)-binding Rossmann-fold superfamily protein
Peaxi162Scf00160g01753.1 NAD(P)-binding Rossmann-fold superfamily protein
Peinf101Scf02877g01053.1 NAD(P)-binding Rossmann-fold superfamily protein
Peaxi162Scf00083g00619.1 NAD(P)-binding Rossmann-fold superfamily protein
Peinf101Scf01063g05016.1 NAD(P)-binding Rossmann-fold superfamily protein
Peaxi162Scf00003g05029.1 NAD(P)-binding Rossmann-fold superfamily protein
XP 019254447.1 PREDICTED: uncharacterized protein LOC109233131 isoform X1 Nicotiana attenuata
XP 019254448.1 PREDICTED: uncharacterized protein LOC109233131 isoform X2 Nicotiana attenuata
XP 019254449.1 PREDICTED: uncharacterized protein LOC109233133 isoform X1 Nicotiana attenuata
XP 019254450.1 PREDICTED: uncharacterized protein LOC109233133 isoform X2 Nicotiana attenuata
XP 016470339.1 PREDICTED: 3-oxoacyl-acyl-carrier-protein reductase FabG-like Nicotiana tabacum
XP 009773767.1 PREDICTED: 3-oxoacyl-acyl-carrier-protein reductase chloroplastic-like Nicotiana sylvestris
XP 009596119.1 3-oxoacyl-acyl-carrier-protein reductase FabG-like Nicotiana tomentosiformis
XP 016442777.1 PREDICTED: 3-oxoacyl-acyl-carrier-protein reductase FabG-like Nicotiana tabacum
XP 009778773.1 PREDICTED: 3-oxoacyl-acyl-carrier-protein reductase chloroplastic-like Nicotiana sylvestris
XP 009593495.1 3-oxoacyl-acyl-carrier-protein reductase FabG-like Nicotiana tomentosiformis
XP 016476213.1 PREDICTED: 3-oxoacyl-acyl-carrier-protein reductase FabG-like Nicotiana tabacum
XP 016540579.1 PREDICTED: 3-oxoacyl-acyl-carrier-protein reductase FabG-like Capsicum annuum
KAF3630452.1 hypothetical protein FXO37 28469 Capsicum annuum
PHT94957.1 hypothetical protein T459 02839 Capsicum annuum
PHT59900.1 hypothetical protein CQW23 02263 Capsicum baccatum
XP 004249129.1 uncharacterized protein LOC101265973 Solanum lycopersicum
XP 027768090.1 uncharacterized protein LOC107001606 Solanum pennellii
TMW92893.1 hypothetical protein EJD97 012433 Solanum chilense
XP 015159580.1 PREDICTED: 3-oxoacyl-acyl-carrier-protein reductase FabG-like Solanum tuberosum
Peinf101Scf01294g05022.1 Unknown protein
XP 009607267.1 3-oxoacyl-acyl-carrier-protein reductase FabG-like Nicotiana tomentosiformis
PHT25915.1 hypothetical protein CQW23 34469 Capsicum baccatum
PHU06158.1 hypothetical protein BC332 26980 Capsicum chinense
XP 016543953.1 PREDICTED: 3-oxoacyl-acyl-carrier-protein reductase FabG-like Capsicum annuum
α-subunit
PhBSβ
Peaxi162Scf00776g00122.1
Peinf101Scf03171g02027.1
XP 016468826.1 PREDICTED: levodione reductase-like Nicotiana tabacum
XP 009618143.1 levodione reductase Nicotiana tomentosiformis
XP 019267113.1 PREDICTED: uncharacterized protein LOC109244473 Nicotiana attenuata
XP 009798974.1 PREDICTED: peroxisomal 24-dienoyl-CoA reductase Nicotiana sylvestris
KAF3618610.1 putative CONSTANS interacting protein 6 Capsicum annuum
KAF3635928.1 putative CONSTANS interacting protein 6 Capsicum annuum
XP 016580500.1 PREDICTED: dehydrogenase/reductase SDR family member 4 Capsicum annuum
PHT43044.1 hypothetical protein CQW23 17069 Capsicum baccatum
XP 006363009.1 PREDICTED: dehydrogenase/reductase SDR family member 4 Solanum tuberosum
AFX67031.1 short-chain alcohol dehydrogenase partial Solanum tuberosum
TMW99131.1 hypothetical protein EJD97 002995 Solanum chilense
XP 004243555.1 uncharacterized protein LOC101255117 Solanum lycopersicum
XP 015082423.1 uncharacterized protein LOC107026087 Solanum pennellii
β-subunit
0.05

Supplementary Figure 4 | Phylogenetic analysis of benzaldehyde synthase subunits from Solanaceae family. The evolutionary history was inferred using the Neighbor-Joining method. The optimal tree with the sum of branch length = 6.0 is shown. The percentage of replicate trees in which the associated taxa clustered together in the bootstrap test (1000 replicates) are shown next to the branches. The tree is drawn to scale, with branch lengths in the same units as those of the evolutionary distances used to infer the phylogenetic tree. The evolutionary distances were computed using the p-distance method and are in the units of the number of amino acid differences per site. The analysis involved 129 amino acid sequences (Supplementary Data 2).

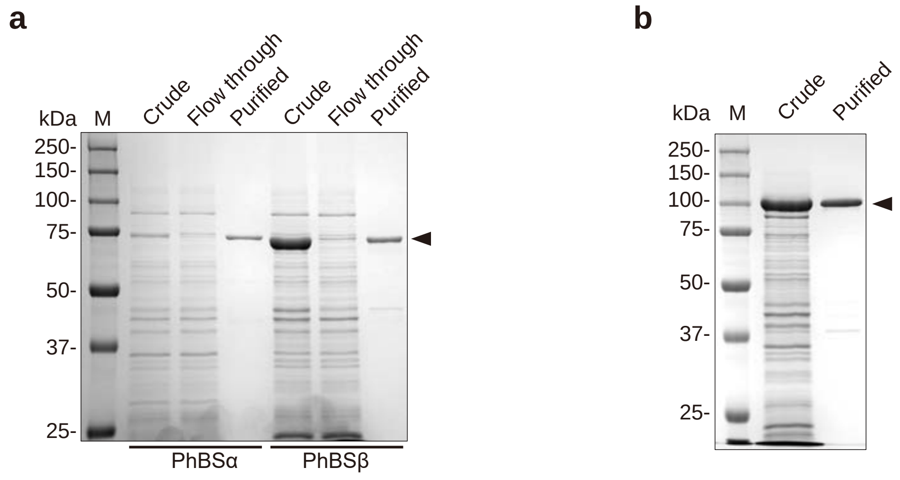

Supplementary Figure 5 | SDS-PAGE gel showing purification of benzaldehyde synthase subunits and Ph4CL1 from *E. coli*. (**a**) Aliquots of fractions from prokaryotic expression and purification of MBP-tagged PhBS subunits. Crude, total soluble bacterial lysate after IPTG induction; Flow through: fractions passed through amylose resin; Purified, ~ 2 μg of purified protein after elution with 10 mM maltose solution. The experiment was repeated at least six times with similar results. (**b**) Purification of Ph4CL1. Crude, total soluble bacterial lysate after IPTG induction; Purified, ~ 2 μg of purified protein after elution with maltose solution. The experiment was repeated three times with similar results. M, protein molecular weight standards. Triangle indicates the position of MBP-tagged PhBS subunits (**a**) and Ph4CL1 protein (**b**). MBP is ~ 42.5 kDa in size.

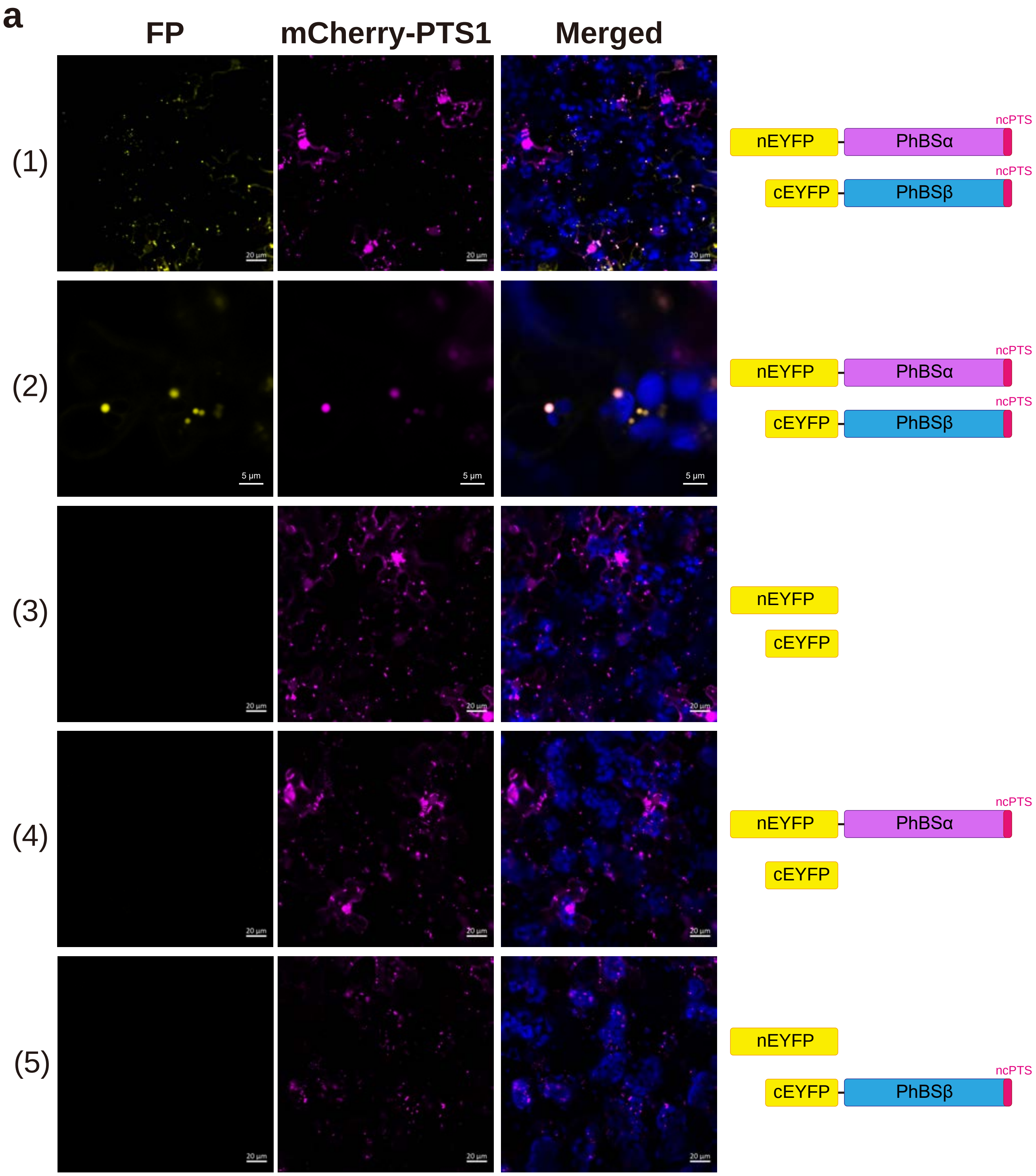
a
FP
mCherry-PTS1
Merged
(1)
(2)
(3)
(4)
(5)
20 µm
5 µm
ncPTS
nEYFP
PhBSα
ncPTS
cEYFP
PhBSβ
ncPTS
nEYFP
PhBSα
ncPTS
cEYFP
PhBSβ
nEYFP
cEYFP
ncPTS
nEYFP
PhBSα
cEYFP
nEYFP
ncPTS
cEYFP
PhBSβ

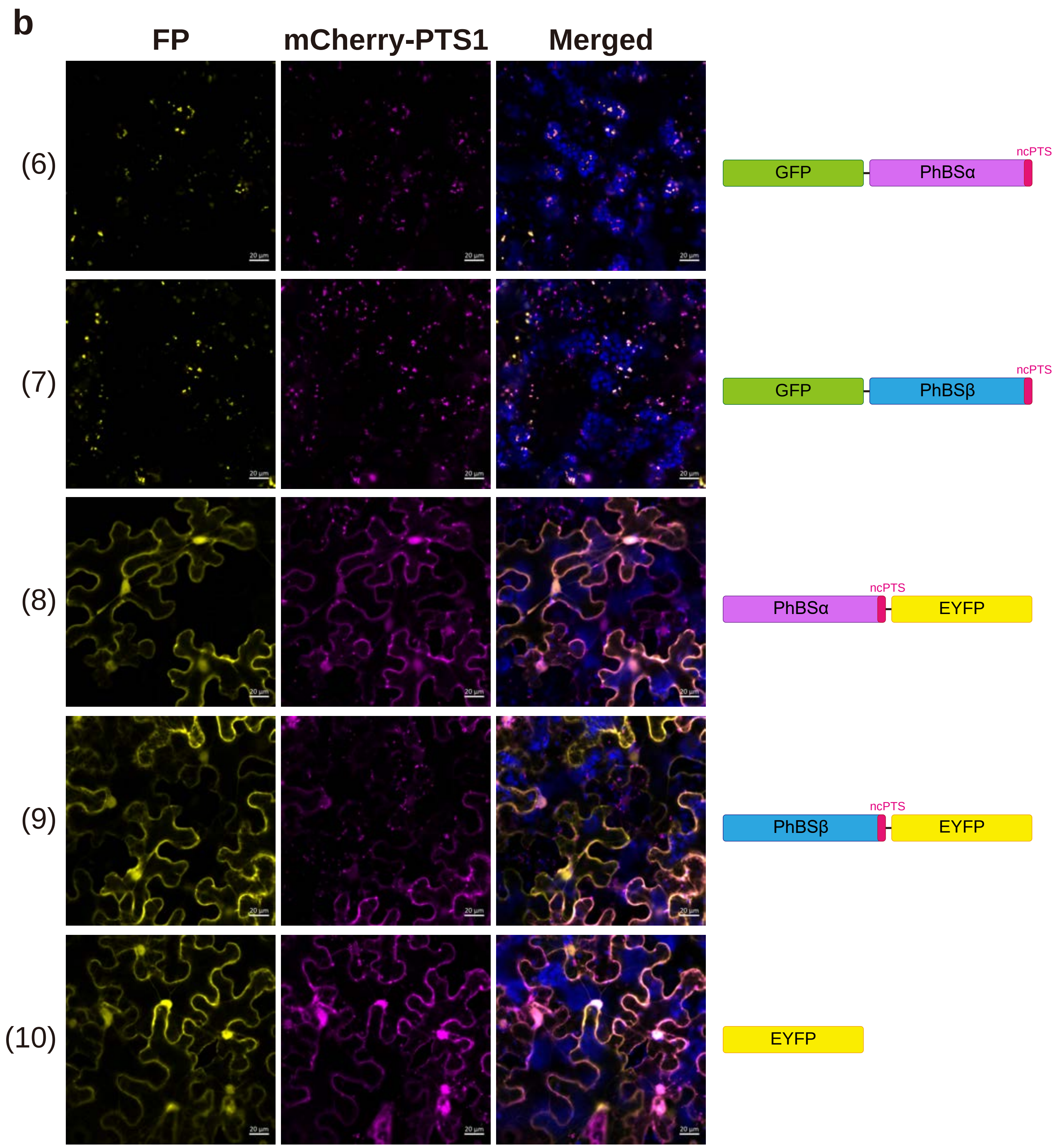
b
FP
mCherry-PTS1
Merged
(6)
(7)
(8)
(9)
(10)
ncPTS
GFP
PhBSα
ncPTS
GFP
PhBSβ
ncPTS
PhBSα
EYFP
ncPTS
PhBSβ
EYFP
EYFP

Supplementary Figure 6 | BiFC detection of protein-protein interactions between PhBS subunits and their subcellular localization. (**a**) Transient BiFC analysis of PhBSα-PhBSβ interaction in *N. benthamiana* leaves detected by confocal laser scanning microscopy. The "FP" panels (yellow) represent signals of reconstituted EYFP as a result of protein-protein interactions; the "mCherry-PTS1" panels (magenta) represent signal of peroxisome-targeted mCherry marker protein; the "Merged" panels show merged EYFP and mCherry signals. Blue signals in the "Merged" panels represent chlorophyll autofluorescence. Schematic diagrams of the protein topologies and combinations for each experiment are illustrated on the right as follows (from amino terminus to carboxyl terminus): (1) nEYFP-PhBSα + cEYFP-PhBSβ; (2) nEYFP-PhBSα + cEYFP-PhBSβ captured with 63× oil immersion objective lens; (3) nEYFP + cEYFP; (4) nEYFP-PhBSα + cEYFP; (5) nEYFP + cEYFP-PhBSβ. The experiment was repeated three times with similar results. (**b**) Subcellular localization of PhBS subunits. PhBS fusion constructs were expressed in *N. benthamiana* leaves and their corresponding transient expression was detected by confocal laser scanning microscopy. The "FP" panels (yellow) represent signals of PhBS fused fluorescent proteins; the "mCherry-PTS1" panels (magenta) represent signal of peroxisome-targeted mCherry marker protein; the "Merged" panels show merged FP and mCherry signals. Blue signals in the "Merged" panels represent chlorophyll autofluorescence. Schematic diagrams of the BS-FP fusion proteins for each experiment are illustrated on the right as follows (from amino terminus to carboxyl terminus): (6) GFP-PhBSα; (7) GFP-PhBSβ; (8) PhBSα-EYFP; (9) PhBSβ-EYFP; (10) EYFP. FP, fluorescent protein; PTS1, classic peroxisomal targeting signal; ncPTS, non-classic PTS. The experiment was repeated three times with similar results. All images were captured using 20× objective lens except for (2). Scale bars = 20 μm, except for (2), in which bars are 5 μm.

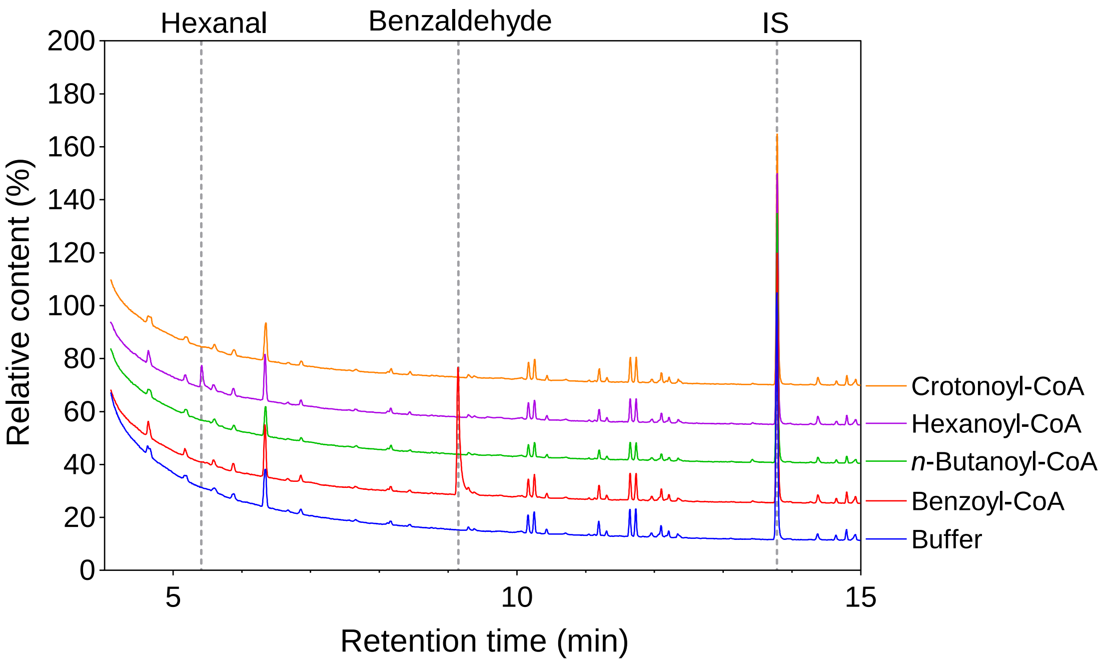

Supplementary Figure 7 | Substrate specificity of purified PhBS. GC-MS analysis of products formed by MBP-tagged PhBS from different short-chain fatty acyl-CoA substrates. Purified PhBS (1:1 ratio between α and β subunits) was incubated with 200 μM benzoyl-CoA or 1 mM fatty acyl-CoA including *n*-butanoyl-CoA, hexanoyl-CoA, and crotonoyl-CoA. All reactions were carried out at 28°C for 1 hour. Shown are total ion currents (TICs) of scan mode (*m*/*z* 35 to 250). The response of internal standard in each run was set as 100%.

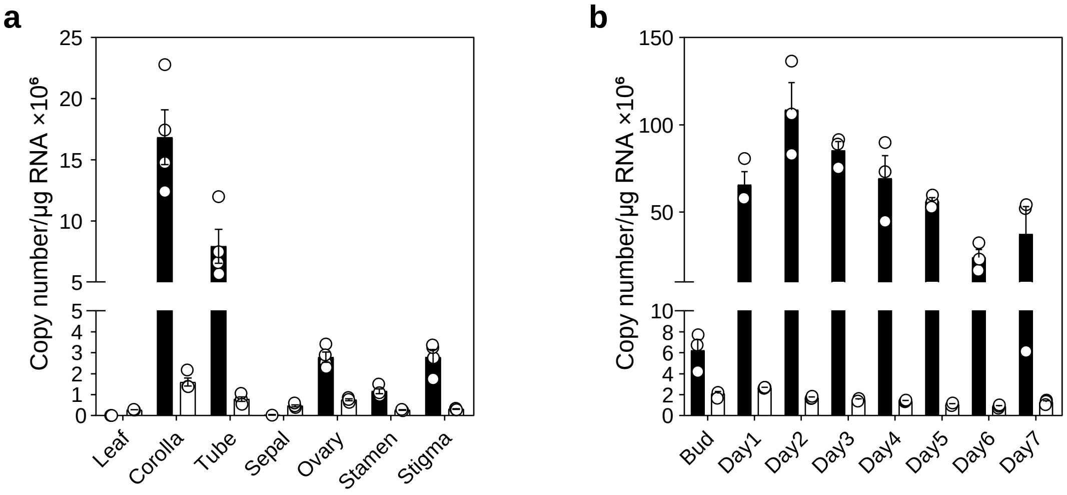

Supplementary Figure 8 | Spatial and developmental expression of *PhBSα* and *PhBSβ* in petunia flowers. (**a**) Tissue-specific expression of *PhBSα* (black bar) and *PhBSβ* (white bar). (**b**) Developmental expression of *PhBSα* (black bar) and *PhBSβ* (white bar) in petunia corolla from mature buds to day 7 post-anthesis. The *PhBSα* and *PhBSβ* expression was determined by qRT-PCR with gene-specific primers and expressed as copy number of transcripts per microgram of total RNA. Shown are means ± SE (*n*=4 biological replicates for **a**; *n*=3 biological replicates for **b**).

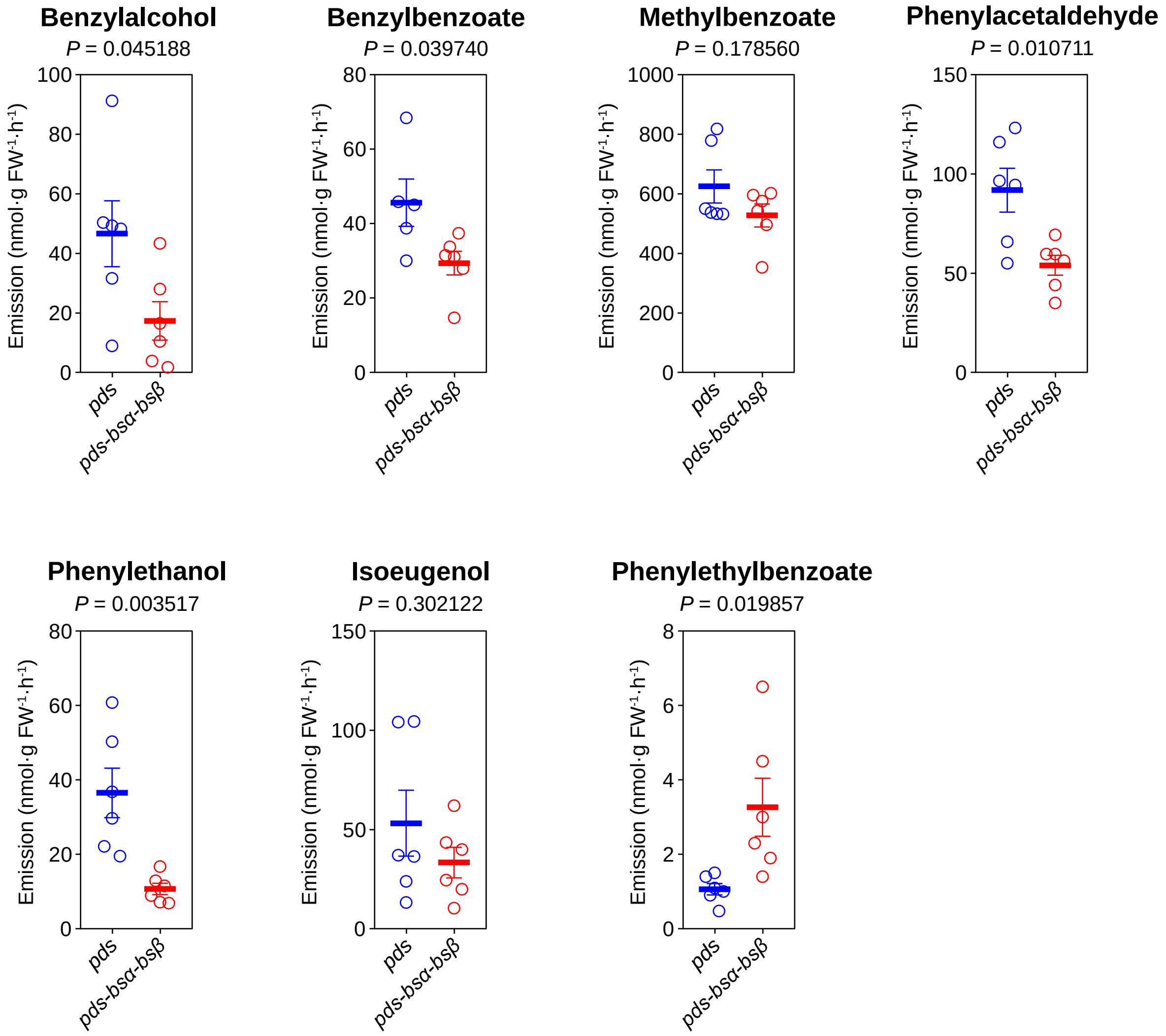

Supplementary Figure 9 | Effect of *PhBS* downregulation on emission of individual petunia volatiles. Emission rates of individual VOCs are shown in *pds* (control) and *pds-bsα-bsβ* VIGS petunia flowers. Volatiles were collected from 2-day-old flowers from 20:00 till 21:00. Data are means ± SE (*n*=6 biological replicates). *P* values, shown on top of each graph, were determined by unpaired two-tailed Student's *t*-test relative to *pds* control.

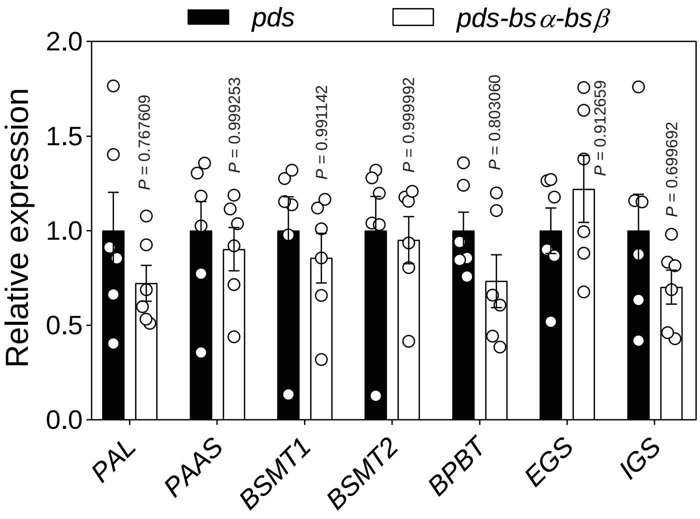

Supplementary Figure 10 | Effect of *PhBS* downregulation on expression of scent biosynthetic genes in petunia flowers. Transcript levels were determined by qRT-PCR with gene-specific primers in *pds* (control) and *pds-bsα-bsβ* VIGS flowers at 21:00 and presented relative to the corresponding levels in *pds* control, set as 1. Data are means ± SE (n=6 biological replicates). P values were determined by two-way ANOVA multiple comparisons test relative to the *pds* controls. Displayed gene identifiers encode the following proteins[1-5]: *BPBT*, benzoyl-CoA:benzyl alcohol/2-phenylethanol benzoyltransferase; *BSMT*, benzoic acid/salicylic acid carboxyl methyltransferase; *EGS*, eugenol synthase; *IGS*, isoeugenol synthase; *PAAS*, phenylacetaldehyde synthase; *PAL*, phenylalanine ammonia lyase.

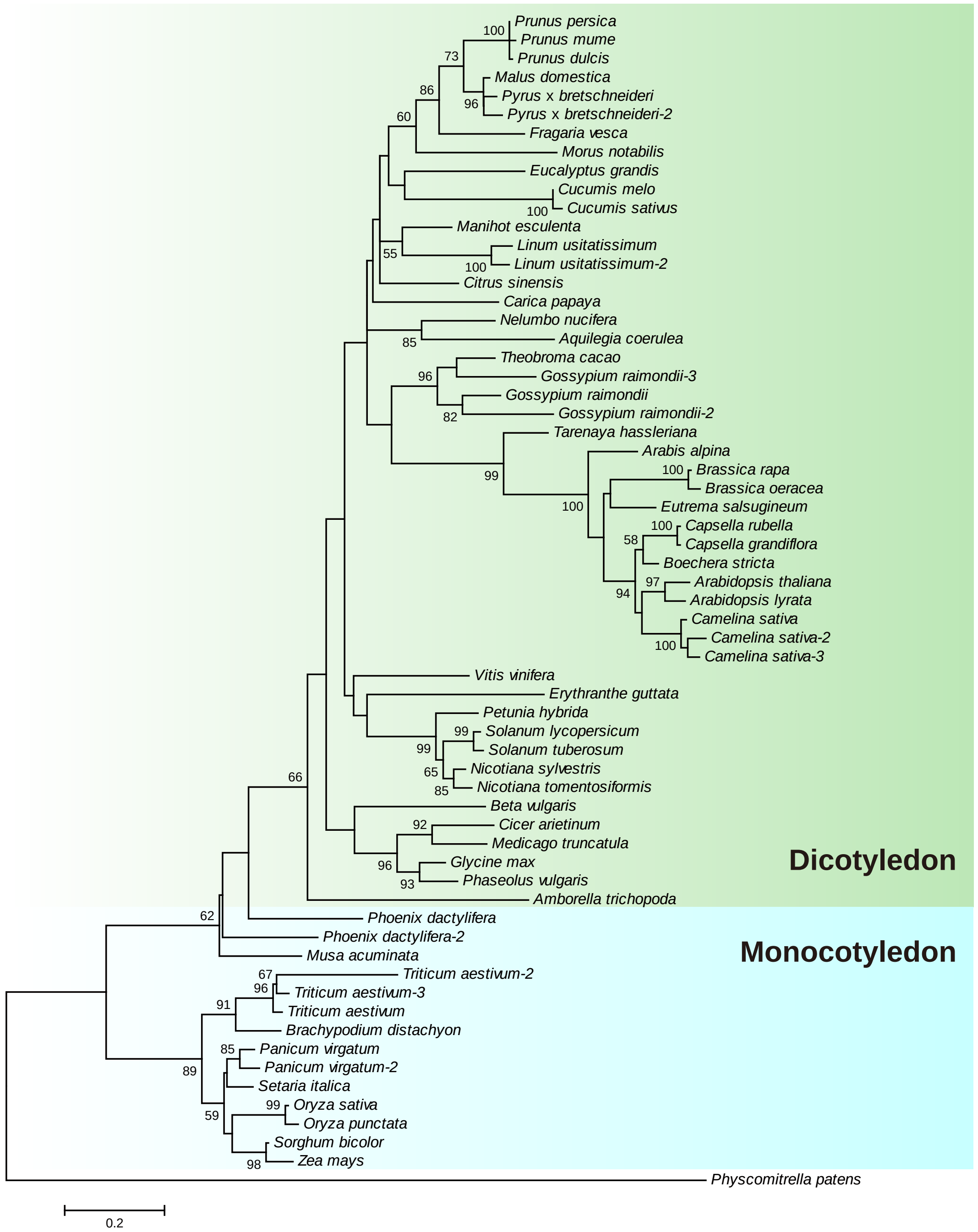

Supplementary Figure 11 | Phylogenetic analysis of BSβ homologs in land plants. The evolutionary history was inferred by using the Maximum Likelihood method based on the JTT matrix-based model. The tree with the highest log likelihood (-7504.55) is shown. The percentage of trees in which the associated taxa clustered together is shown next to the branches. Initial tree(s) for the heuristic search were obtained automatically by applying Neighbor-Join and BioNJ algorithms to a matrix of pairwise distances estimated using a JTT model, and then selecting the topology with superior log likelihood value. A discrete Gamma distribution was used to model evolutionary rate differences among sites (8 categories (+G, parameter = 1.9404)). The tree is drawn to scale, with branch lengths measured in the number of substitutions per site. The analysis involved 63 amino acid sequences.

a

```
PhBSα -MEKQTQ-ARFLEPWRDLKGKVVMVTGASSGIGLDLCLDLAKAGCKIIAAARRVDRLKSLCNQIN--------AAADDDD 70
AtBSα MSNHQTQVLKLLEPWCELKDKVVLVTGASSGIGREICLDLAKAGCQVIAAARRVDRLNSLCSEIN--------SFSSTG- 71
PdBSα ---MANQVADHLEPWRHLHGKVVMVTGASSGLGREFCLDLAKAGCRIVAAARRIDRLQSVCDEINQLTVTAPSSSSSSSS 77
SlBSα -METHAKSVRQLEPWGELAGKVVMVTGASSGIGRDFCSDLSKAGCRIIAAARRIDRLQSLCDEIN--------SNSSNGS 71

PhBSα DNN----RAVAVELDVSANGDAIEAAVKEAWGAFGRIDALVNNAGVRGTVHSPLELTEEECHNTFRTNLTGTWLVSKYVC 146
AtBSα ------IQAAALELDVSSDAATIQKAVREAWDIFGKIDALINNAGIRGNVKSSLDLSEDEWDNVFKTNLKGPWLVSKHVC 145
PdBSα VNGPGSSRAVAVELDISADGPAIEKSVNKAWDAFGRIDALVNNAGVRGNVSSSLELSEEEWNNVFKTNLTGSWLVSKYVS 157
SlBSα TKSSQDLRAVAIELDVSANGSAIEAAVQKAWDAFGRIDGLVNNAGFRGSVLSPLELSEEEWEKIHKTNLRGAWLVTKYVC 151

PhBSα KFMRDAKQGGSVINISSIAGLNRGQLPGGLAYVSSKGAVNMVTKVMALELGVDKIRVNSISPGLFKSEITQGLIQKEWLN 226
AtBSα MLMRDAKRGGSVINISSIAGI-RGMLPGGLAYACSKGGVDTMSRMMALELGVHKIRVNSIAPGLFKSEITQGLMQKEWLK 224
PdBSα IRMRDADQGGSIINVSSIAGLHRGYLPGAVAYNCSKAGVNTLAKVMAMELGVHKIRVNAISPGLFRSEITEGLMKKDWLH 237
SlBSα MHMRAANQGGSIINISSIAGINRGQLPGSLAYTSSKEALNSITKVLALELGPYKIRVNSISPGLFKSEITEGLIQKDWIK 231

PhBSα NVAMRTIPLRTHGNSADPALTSVVRYLIHDSSEYVSGNLFIVDAGATLPGVPIFSSL 283
AtBSα NVTERTVPLKVQQ-TVDPGLTSLVRYLIHDSSQYISGNTYIVDSGATLPGVPIFSSL 280
PdBSα NVAMKTVPLRTFG-TSDPALTSLVRYLVHDSSEYVSGNVYIVDAGATLPGVPIFSAL 293
SlBSα NIELRTIPLRTHG-TSHPALTSVVRYLIHDSSEYVSGNMFIVDAGATLPGVPIFSSL 287
```

b

```
PhBSβ MEN-SGKKVLLTSNGDDICNNIAYHLAQRGCQLVLMGNESQLKSVAEMIKQSLGGSAAVEVVGLDMEEDREAAFEEAVDK 79
AtBSβ MEN-PAKRVLMTSNGDEVSRNIAFHLAKHGCKLVMMGNEGSLRSIVDKIRDSIEGAFPADVIALDMESDSEVAFHAAVQK 79
PdBSβ MGTSPGKKVLLTSNGDAISHNIAFSLAQRGCRLVLMGKESCLRSIQQKIKGSLEGVVPVEVVDVDMEDKREGAFDEAVDK 80
SlBSβ MEN-PGKKVLLTSNGDEICNNIAYHLAQRGCQLVLMGNERQLKSVAENIKQSLKGSVAVEVVGLDMTEDRETAFDEAVDK 79

PhBSβ AWKIFGKLDALVNCYSYEGKMQDPLQLIEEEFKKIVKINFMAGWYLMKCISKKMRDDKSGGSIVFLTSIIGAERGIYQGA 159
AtBSβ AWELSGHFDAFLNSYTYQGKVQDILQVSQDEFHRITKINLTAPWFLLKAVATRMKDHGSGGSIVFMATIASGERALYPGA 159
PdBSβ ACHILGNLDAFVHCYTYEGKMQEHLELAEDEFKKIMKRNFMSAWFLLNAVGRRMRDYKSGGSIIFLTSIIGAERGIYPGA 160
SlBSβ AWKIFGKLDALVHCYAYEGKMQDPLQLIDDDEFKKIVKINFMAGWYLLKCIGNRMRDGKSGGSIVFMTSIIGAERGIYQGA 159

PhBSβ AAYGSCLGGIQQLVRLAAIELGKHQIRVNGISRGLHLQDEYPKAVGLERAEKMTKEAAPLNRWLDVKKDLASTVIYLISD 239
AtBSβ DAYASTSAAIHQLVRASAMSLGKHKIRVNMISRGLHLDDEYTASVGRDRAQKLVKDAAPLGQWLNPDTDLYSTVIYLISD 239
PdBSβ AANSACSAAVQQLARTSALEIGRYQIRVNAIARGLHLEDEYPMFVGMERAKKLVKEAAPLQRWLDVKNDLASTVIYLISD 240
SlBSβ AAYGSCAAGIQQLVRLSAIELGKYQIRVNGILRGLHLEDEFPLSVGKERAVKLTKEAAPLNRWLDPKKDLASTVIYLISD 239

PhBSβ DSRYMTGTSIFVDGAQSLVRPRMRSYM 266
AtBSβ GSRFMTGTTVLVDGAQSLTRPRLKSYM 266
PdBSβ GSKYMTGTTIFVDGGQSLTRPRMRSYM 267
SlBSβ DSRYMTGTSIFVDGAQSLVRPRMRSYM 266
```

Supplementary Figure 12 | Alignment of deduced amino acid sequences for BS subunits from four species. (**a**) Alignment of amino acid sequences for BSα subunits from *P. hybrida*, *A. thaliana*, *Prunus dulcis* and *S. lycopersicum*. (**b**) Alignment of amino acid sequences for BSβ subunits from petunia, Arabidopsis, almond and tomato. Conserved residues are shaded in black, while similar residues are shaded in dark gray.

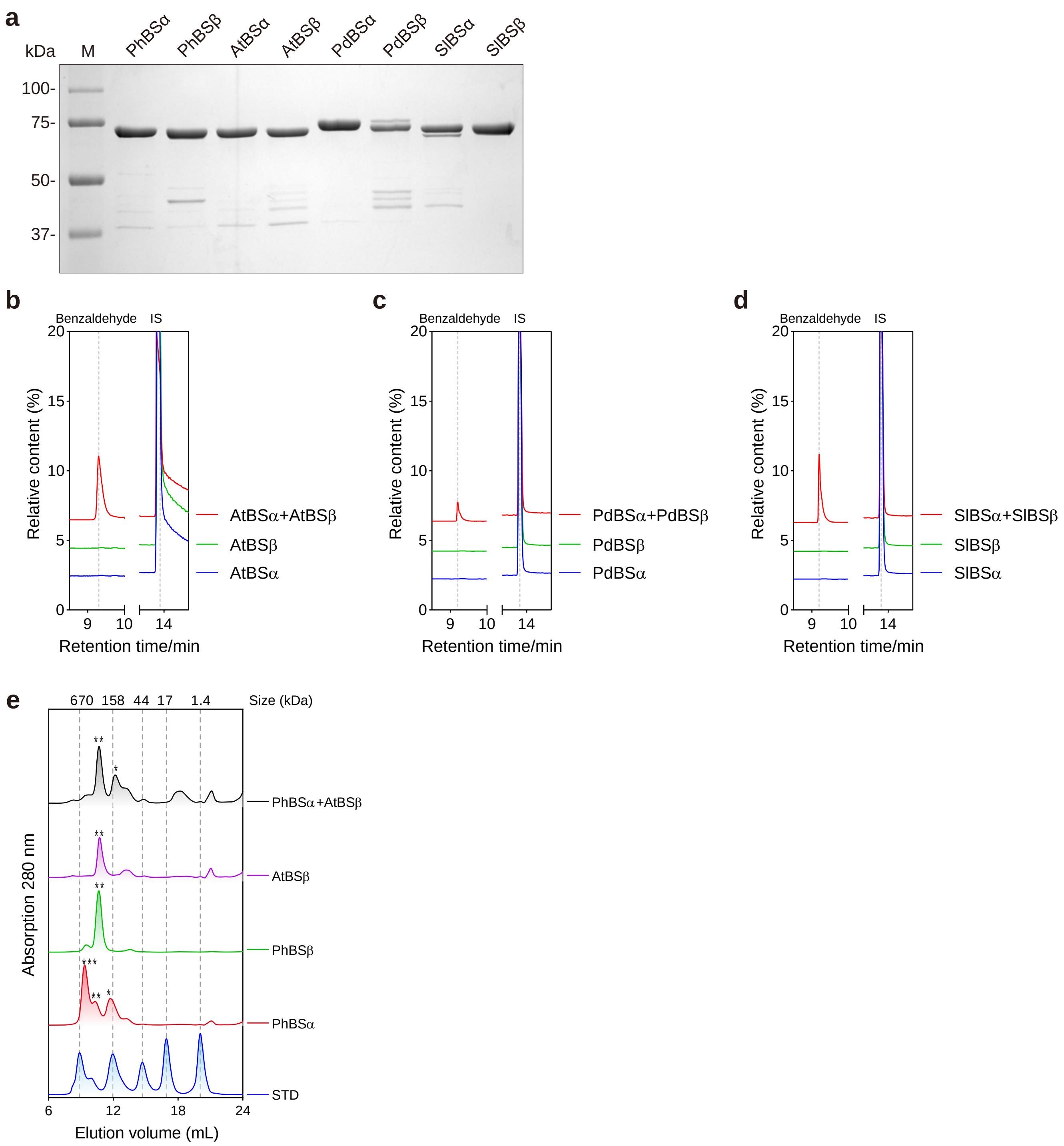

Supplementary Figure 13 | Purification and characterization of BS from phylogenetically distant species. (**a**) SDS-PAGE analysis of ~2 μg of purified MBP-tagged BS subunits from petunia, Arabidopsis, almond and tomato. M, protein molecular weight standards. The experiment was repeated at least six times with similar results. (**b - d**) GC-MS analysis of products formed *in vitro* by Arabidopsis (**b**), almond (**c**) and tomato (**d**) purified recombinant BS proteins in enzymatic assays. The response of internal standard in each run was set as 100%. (**e**) Gel filtration chromatography of purified recombinant PhBS subunits, AtBSβ and PhBSα-AtBSβ hybrid. 500 μL purified proteins (~ 500 μg) were loaded onto a Superdex 200 Increase 10/300 GL size exclusion column and eluted with Phosphate Buffered Saline (PBS). The column was calibrated with following standards: bovine thyroglobulin (670 kDa), bovine gamma globulin (158 kDa), chicken ovalbumin (44 kDa), horse myoglobulin (17 kDa), and vitamin B-12 (1.4 kDa). Peak labels are as follows: *, dimers; **, tetramers; and ***, multimers. STD, chromatogram of calibration standards.

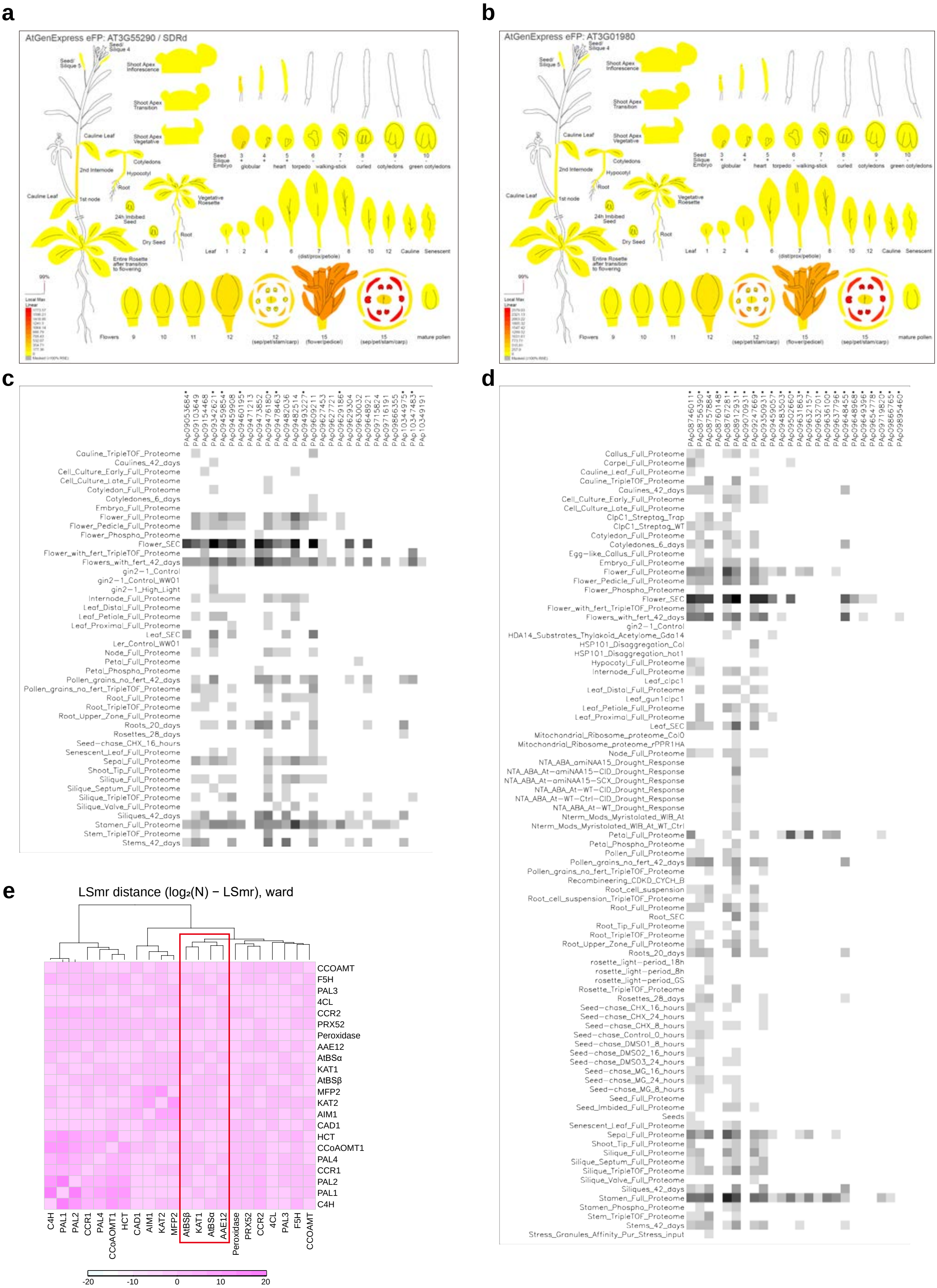
a
AtGenExpress eFP: AT3G55290 / SDRd
b
AtGenExpress eFP: AT3G01980
c
d
e
LSmr distance (log₂(N) − LSmr), ward
CCOAMT
F5H
PAL3
4CL
CCR2
PRX52
Peroxidase
AAE12
AtBSα
KAT1
AtBSβ
MFP2
KAT2
AIM1
CAD1
HCT
CCoAOMT1
PAL4
CCR1
PAL2
PAL1
C4H
-20
-10
0
10
20

Supplementary Figure 14 | Expression profile of *AtBSα* and *AtBSβ*. Tissue-specific and developmental expression levels of *AtBSα* (**a**) and *AtBSβ* (**b**) transcripts. Data in **a** and **b** were obtained from ePlant (https://bar.utoronto.ca/eplant/). Peptide enrichment of AtBSα (**c**) and AtBSβ (**d**) in different tissues. Data in **c** and **d** were retrieved from Arabidopsis PeptideAtlas project website (http://www.peptideatlas.org/builds/arabidopsis/). Column names represent distinct observed peptides of each protein, with asterisk denotes single genome mapping. (**e**) Hierarchical clustering of *AtBS* genes as well as genes involved in β-oxidation and lignin biosynthesis pathways. The plot was generated using ATTED-II Hcluster tool (https://atted.jp/top_draw/#Hcluster). Genes used to build this plot are as follows: *AtBSα* (AT3G55290), *AtBSβ* (AT3G01980), *PAL1* (AT2G37040), *PAL2* (AT3G53260), *PAL3* (AT5G04230), *PAL4* (AT3G10340), *4CL* (AT4G19010), *CCR1* (AT1G15950), *CCR2* (AT1G80820), *C4H* (AT2G30490), *HCT* (AT5G48930), *CAD1* (AT1G72680), *CCoAOMT1* (AT4G34050), *F5H* (AT4G36220), *Peroxidase* (AT5G66390), *PRX52* (AT5G05340), *CCOAMT* (AT1G67980), *KAT1* (AT1G04710), *KAT2* (AT2G33150), *AIM1* (AT4G29010), *MFP2* (AT3G06860), and *AAE12* (AT1G65890). The subcluster containing both *AtBSα* and *AtBSβ* is highlighted with a red box.

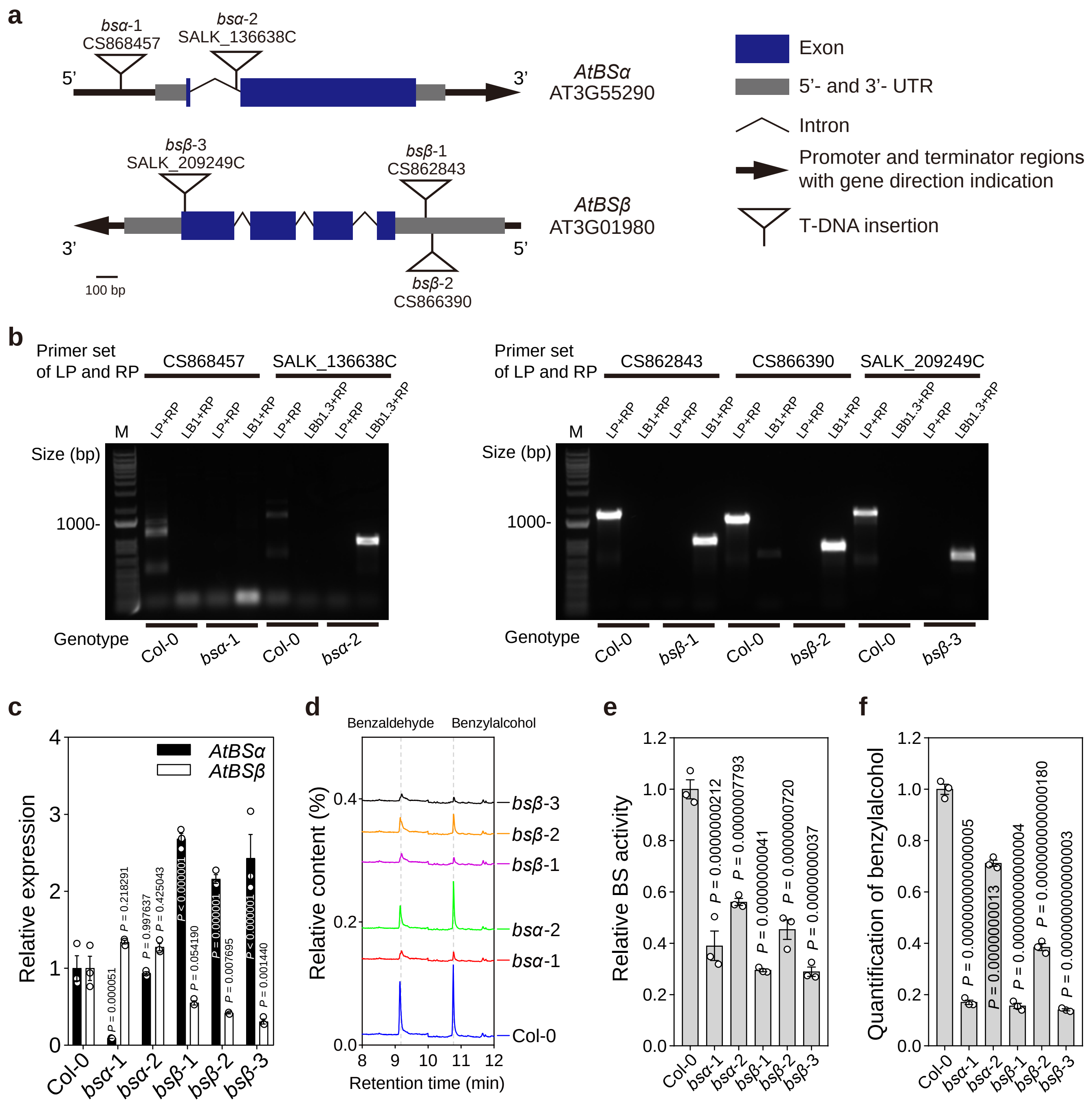

Supplementary Figure 15 | Characterization of Arabidopsis *bs* mutants. (**a**) Schematic diagrams showing the gene structures of *AtBSα* (AT3G55290) and *AtBSβ* (AT3G01980) and T-DNA insertion sites in five *bs* mutant lines. (**b**) Genotyping of the *bs* mutants by PCR analysis of genomic regions flanking each T-DNA insertion and a corresponding region of the wild-type gene. The experiment was repeated three times with similar results. (**c**) qRT-PCR analysis of *AtBSα* and *AtBSβ* transcripts in floral tissues of Arabidopsis wild type (ecotype Col-0) and *bs* mutants using gene-specific primers. Expression of each gene in wild type was set to 1. Data are means ± SE (n=3 biological replicates, each replicate contains > 100 fully opened flowers and flower buds). P values were determined by two-way ANOVA multiple comparisons test of each gene relative to the Col-0 controls. (**d**) GC-MS analysis of BS activities in crude flower extracts from Arabidopsis wild type and bs mutants. The response of internal standard in each run was set to 100%. (**e**) Quantification of apparent BS activities shown in **d**. BS activity in wild type was set to 1. Data are means ± SE (n=3 biological replicates). (**f**) Quantification of benzylalcohol formation *in vitro* assays with crude extracts shown in **d**. Benzylalcohol level produced by a crude extract from wild type was set to 1. Data are means ± SE (n=3 biological replicates). P values in **e** and **f** were determined by ordinary one-way ANOVA multiple comparisons test relative to the wild type controls.

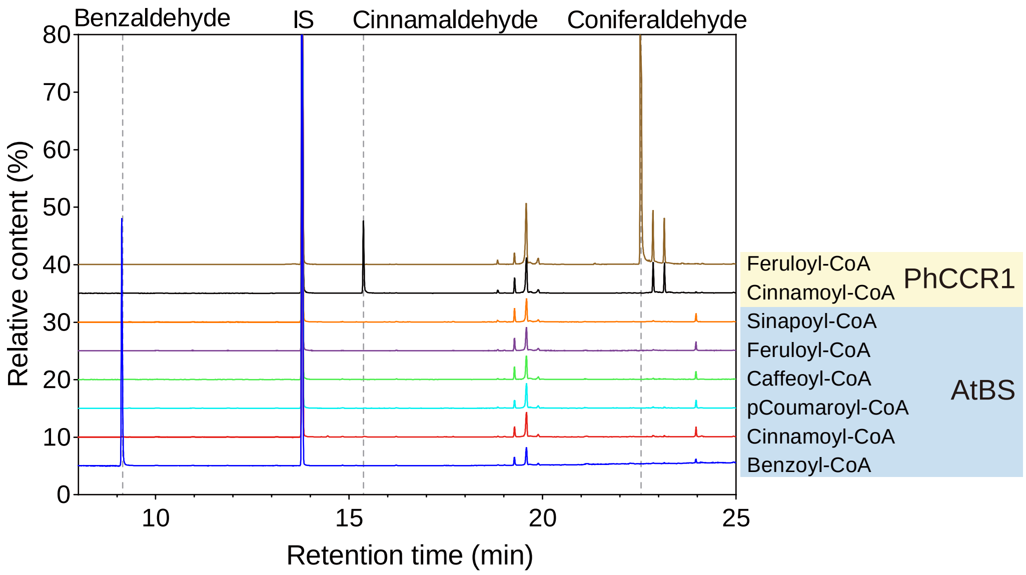

Supplementary Figure 16 | Substrate specificity of purified MBP-tagged AtBS. GC-MS analysis of products formed by AtBS from different hydroxycinnamoyl-CoA substrates. Purified AtBS (1:1 ratio between α and β subunits) was incubated with benzoyl-CoA and its structural analogs including cinnamoyl-CoA, *para*-coumaroyl-CoA, caffeoyl-CoA, feruloyl-CoA, and sinapoyl-CoA. All CoA esters except benzoyl-CoA were synthesized using Ph4CL1 and their corresponding free acids. Formation of cinnamaldehyde and coniferaldehyde by purified PhCCR1 was used as a positive control. Shown are combined EICs of mass units 106 (benzaldehyde), 128 (internal standard), 131 (cinnamaldehyde), and 178 (coniferaldehyde). The response of internal standard in each run was set as 100%.

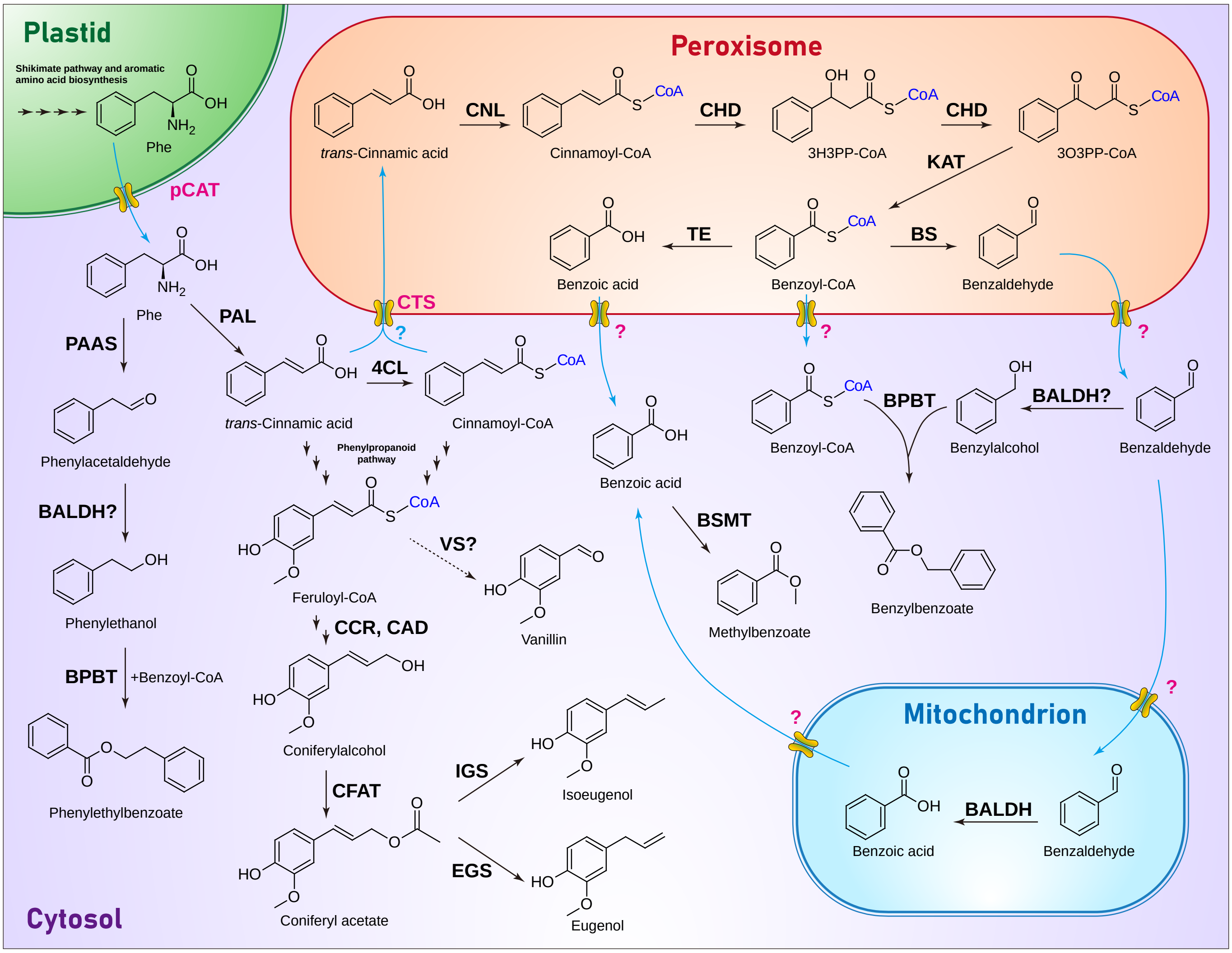

Supplementary Figure 17 | Proposed VOC biosynthetic network in petunia flowers. Enzymes responsible for each biochemical reaction are shown in bold black. Established biochemical reactions are presented by solid black arrows, while unidentified steps are shown by dashed arrows. Transporters involved in metabolite transport between different subcellular locations are shown in bold magenta, with the flow of metabolites indicated by cyan arrows. Cross-organelle transport of metabolites by unknown transporters or diffusion are indicated with magenta question marks. Abbreviations: BALDH, benzaldehyde dehydrogenase; BPBT, benzoyl-CoA:benzyl alcohol/2-phenylethanol benzoyltransferase; BS, benzaldehyde synthase; BSMT, benzoic acid/salicylic acid carboxyl methyltransferase; CAD, cinnamyl alcohol dehydrogenase; CCR, cinnamoyl-CoA reductase; CFAT, coniferyl alcohol acyltransferase; CHD, cinnamoyl-CoA hydratase/dehydrogenase; CNL, cinnamate-CoA ligase; CTS, peroxisomal cinnamic acid/cinnamoyl-CoA transporter COMATOSE. The substrate specificity of CTS is unclear, as indicated by a cyan question mark next to it; EGS, eugenol synthase; IGS, isoeugenol synthase; KAT, 3-ketoacyl thiolase; PAAS, phenylacetaldehyde synthase; PAL, phenylalanine ammonia lyase; pCAT, plastidial cationic amino acid transporter; Phe, phenylalanine; 3H3PP-CoA, 3-hydroxy-3-phenylpropanoyl-CoA; 3O3PP-CoA, 3-oxo-3-phenylpropanoyl-CoA; TE, thioesterase; VS, vanillin synthase.

Supplementary references

1. Boatright, J. *et al.* Understanding *in vivo* benzenoid metabolism in petunia petal tissue. *Plant Physiol.* **135**, 1993–2011 (2004).
2. Kaminaga, Y. *et al.* Plant phenylacetaldehyde synthase is a bifunctional homotetrameric enzyme that catalyzes phenylalanine decarboxylation and oxidation. *J. Biol. Chem.* **281**, 23357–23366 (2006).
3. Negre, F. *et al.* Regulation of methylbenzoate emission after pollination in snapdragon and petunia flowers. *Plant Cell* **15**, 2992–3006 (2003).
4. Koeduka, T. *et al.* Eugenol and isoeugenol, characteristic aromatic constituents of spices, are biosynthesized via reduction of a coniferyl alcohol ester. *Proc. Natl. Acad. Sci. U. S. A.* **103**, 10128–10133 (2006).
5. Koeduka, T. *et al.* The multiple phenylpropene synthases in both *Clarkia breweri* and *Petunia hybrida* represent two distinct protein lineages. *Plant J.* **54**, 362–374 (2008).

Supplementary Table 1. Primers used in this research

Primer name	Sequence (5' to 3')
**For VIGS**	
PhBSα>pTRV2-PDS/F	ATCCGGTACCGAGCTCGGAGAAGCAAACTCAGGCACG
PhBSα>pTRV2-PDS/R	AGAATTTTCCTCCAAAAGCACCCC
PhBSβ>pTRV2-PDS/F	TGCTTTTGGAGGAAAATTCTGGAAAGAAG
PhBSβ>pTRV2-PDS/R	CGAGACGCGTGAGCTCTTGCATTTTTCCTTCATATGAATAGCAGTTTACC
**For heterologous expression of BS**	
PhBSα>pMAL/F	GCGATATCGTCGACGGATCCATGGAGAAGCAAACTCAGGCAC
PhBSα>pMAL/R	CCTGCAGGGAATTCGGATCCCTACAGGGATGAGAAAATCGGGACAC
PhBSβ>pMAL/F	GCGATATCGTCGACGGATCCATGGAAAATTCTGGAAAGAAGGTGTTG
PhBSβ>pMAL/R	CCTGCAGGGAATTCGGATCCTCACATGTATGAGCGCATTCGC
PhBSβ>pET32b/F	CTTTAAGAAGGAGATATACATATGGAAAATTCTGGAAAGAAGGTGTTGC
PhBSβ>pET32b/R	TCGAGTGCGGCCGCAAGCTTCATGTATGAGCGCATTCGCG
AtBSα>pMAL/F	GCGATATCGTCGACGGATCCATGAGCAATCATCAAACTCAGGTGT
AtBSα>pMAL/R	CCTGCAGGGAATTCGGATCCTCAGAGAGATGAAAAGATAGGCACACCG
AtBSβ>pMAL/F	GCGATATCGTCGACGGATCCATGGAAAATCCGGCGAAGAGAG
AtBSβ>pMAL/R	CCTGCAGGGAATTCGGATCCTCACATGTAGGATTTGAGACGGGGT
**For Y2H assay**	
PhBSα>pGAD/F	CCATGGAGGCCAGTGAATTCATGGAGAAGCAAACTCAGGCAC
PhBSα>pGAD/R	TGCCCACCCGGGTGGAATTCCTACAGGGATGAGAAAATCGGGACAC
PhBSβ>pGAD/F	CCATGGAGGCCAGTGAATTCATGGAAAATTCTGGAAAGAAGGTGTTG
PhBSβ>pGAD/R	TGCCCACCCGGGTGGAATTCTCACATGTATGAGCGCATTCGC
PhBSα>pGBK/F	TATGGCCATGGAGGCCGAATTCATGGAGAAGCAAACTCAGGCAC
PhBSα>pGBK/R	CGACGGATCCCCGGGAATTCCTACAGGGATGAGAAAATCGGGACAC
PhBSβ>pGBK/F	TATGGCCATGGAGGCCGAATTCATGGAAAATTCTGGAAAGAAGGTGTTG
PhBSβ>pGBK/R	CGACGGATCCCCGGGAATTCTCACATGTATGAGCGCATTCGC
**For subcellular localization and BiFC**	
PhBSα>pCNHP-eYFP/F	TATTCTGCCCAAATTCGCGCCATGGAGAAGCAAACTCAGGCAC
PhBSα>pCNHP-eYFP/R	AGCTCCTCGCCCTTGCTCACCATCAGGGATGAGAAAATCGGGACAC
PhBSβ>pCNHP-eYFP/F	TATTCTGCCCAAATTCGCGCCATGGAAAATTCTGGAAAGAAGGTGTTG
PhBSβ>pCNHP-eYFP/R	AGCTCCTCGCCCTTGCTCACCATCATGTATGAGCGCATTCGCG
PhBSα>pCNHP-nEYFP-C/F	CCACAACATCGAGGACATGGAGAAGCAAACTCAGGCAC
PhBSα>pCNHP-nEYFP-C/R	AAAATTTAATGAAACCAGAGTTAACCATGGCTACAGGGATGAGAAAATCGGGACAC
PhBSβ>pCNHP-nEYFP-C/F	GGACGAGCTGTACAAGATGGAAAATTCTGGAAAGAAGGTGTTG
PhBSβ>pCNHP-nEYFP-C/R	AAAATTTAATGAAACCAGAGTTAACCATGGTCACATGTATGAGCGCATTCGCG
**For pathway reconstitution in tobacco**	
PhCNL>pCNHP/F	CTGCCCAAATTCGCGATGGACGAGTTACCAAAATGTGGAG
PhCNL>pCNHP/R	TTTAATGAAACCAGAGTTAACTACAGACGAGCTGGCAAATCAAG
PhCHD>pCNHP/F	CTGCCCAAATTCGCGATGGCTCAAGTTAAGGTGACGAT
PhCHD>pCNHP/R	TTTAATGAAACCAGAGTTAATTACATGCGTGACCTTGAAGCTG
PhKAT>pCNHP/F	CTGCCCAAATTCGCGATGGAGAAAGCAATTCAAAGGCAAAG
PhKAT>pCNHP/R	TTTAATGAAACCAGAGTTAATTATTTCGCATCCTTGGATAAGAAACTGTTG
PhPAL1>pCNHP/F	CTGCCCAAATTCGCGATGGAGTATGCCAATGAAAACTGTAATGG
PhPAL1>pCNHP/R	TTTAATGAAACCAGAGTTAATTAGCAGAGTGGAAGAGGAGCAC
PhBSα>pCNHP/F	CTGCCCAAATTCGCGATGGAGAAGCAAACTCAGGCACGTTTC
PhBSα>pCNHP/R	TTTAATGAAACCAGAGTTAACTACAGGGATGAGAAAATCGGG
PhBSβ>pCNHP/F	CTGCCCAAATTCGCGATGGAAAATTCTGGAAAGAAGGTGTTGCTTACT
PhBSβ>pCNHP/R	TTTAATGAAACCAGAGTTAATCACATGTATGAGCGCATTCGC
**For qRT-PCR analysis of gene expression**	
AtACT2/F	TGGCTGAGGCTGATGATATTCAACCAATCG
AtACT2/R	CCAACATATGCATCCTTCTGGTTCATCCCA
AtBSα/F	CCCATTGGCCATCGAACACACCACAC
AtBSα/R	AGCTGCGGCTTGGATTCCAGTTGAAC
AtBSβ/F	GGAAAATCCGGCGAAGAGAGTGTTGATG
AtBSβ/R	TGCTATAACATCGGCAGGGAAGGCTCC
PhEF1α/F	CCTGGTCAAATTGGAAACGG
PhEF1α/R	CAGATCGCCTGTCAATCTTGG
PhBSα/F	CACTCTCCGTTGGAATTGACAGAGGAGGAG
PhBSα/R	CCACCTGGTAATTGCCCACGATTAAGACC
PhBSβ/F	CTTGACCTCAATTATTGGTGCGGAGAGAGG
PhBSβ/R	TCCTGGAGGTGCAAGCCACGAGATATG
BPBT/F	CCATTAGTTTTCTATTGTTACATGGC
BPBT/R	CACTCGACAATACAAAAGACACC
BSMT1/F	AAGGCACTCAATGTCTATTTTCGG
BSMT1/R	GAATAAGATAATTCAATATGTGCGTGAAC
BSMT2/F	TGCACTTCATAGGTGGTCGAG
BSMT2/R	AGAGAGATCTGAAAGGACCCCT
PAL/F	ACTCTGTTAATGACAACCCCTTGAT
PAL/R	CTGAAAATTCCCACCATGCA
EGS/F	ATCGCAACATCTCCAATTCCA

EGS/R	CAAACGATGGCTCGATCACA
IGS/F	TGATGCAAAGGCTGTGTTGAA
IGS/R	TTGCTGCCCTTGGATCATCT
**For verification of Arabidopsis T-DNA insertion lines**	
LB1	GCCTTTTCAGAAATGGATAAATAGCCTTGCTTCC
LBb1.3	ATTTTGCCGATTTCGGAAC
SAIL_1152_B06/LP	ACATGTCACAATGATTTCGTATCC
SAIL_1152_B06/RP	CCCTCAATGGAATCTCTAATCTTG
SAIL_198_H07/LP	ACATGTCACAATGATTTCGTATCC
SAIL_198_H07/RP	GGAGGAAAGAAGACAAAATGAGAG
SALK_209249/LP	GATTAAGTACGTACCTGGGGAATG
SALK_209249/RP	TATGTTTGTTGATGATTGGGTTTC
SAIL_840_G05.C/LP	ATATGTTTGGTATTTGTTGATGCG
SAIL_840_G05.C/RP	GCTCATATTGTTTTCTCTGTGTGG
SALK_136638.19.15.x/LP	TCGGTATTATTGAACCATTGATTG
SALK_136638.19.15.x/RP	TGATGAAACGTCTAACTCAAGAGC

F: forward primer; R: reverse primer.
