# Supplementary material for: A peroxisomal heterodimeric enzyme is involved in benzaldehyde synthesis in plants
Source: Nat Commun. 2022 Mar 15;13:1352. doi: 10.1038/s41467-022-28978-2 (PMC8924275; doi:10.1038/s41467-022-28978-2)
Supplement: Supplementary file 2 — Description of Additional Supplementary Files [file 41467_2022_28978_MOESM2_ESM.pdf]

Description of Additional Supplementary Files

File name: Supplementary Data 1

Description: List of all detected proteins in four analyzed fractions after partial purification of PhBS.

File name: Supplementary Data 2

Description: Amino acid sequences used to generate Supplementary Figure 4.
